# Blood ACE Phenotyping for Personalized Medicine: Revelation of Patients with Conformationally Altered ACE

**DOI:** 10.3390/biomedicines11020534

**Published:** 2023-02-13

**Authors:** Sergei M. Danilov, Mark S. Jain, Pavel A. Petukhov, Olga V. Kurilova, Valery V. Ilinsky, Pavel E. Trakhtman, Elena L. Dadali, Larisa M. Samokhodskaya, Armais A. Kamalov, Olga A. Kost

**Affiliations:** 1 Department of Medicine, Division of Pulmonary, Critical Care, Sleep and Allergy, University of Illinois, Chicago, IL 60607, USA; 2 Department of Medicine, University of Arizona Health Sciences, Tucson, AZ 85721, USA; 3 Medical Center, Lomonosov Moscow State University, 119992 Moscow, Russia; 4 Department of Pharmaceutical Sciences, College of Pharmacy, University of Illinois, Chicago, IL 60612, USA; 5 Genotek, Ltd., 105120 Moscow, Russia; 6 Dmitry Rogachev National Medical Research Center of Pediatric Hematology, Oncology and Immunology, 117997 Moscow, Russia; 7 Medico-Genetic Center, 115478 Moscow, Russia; 8 Chemistry Faculty, Lomonosov Moscow State University, 119991 Moscow, Russia

**Keywords:** angiotensin I-converting enzyme, blood, conformational changes, plasma ACE, screening, bilirubin, Gilbert syndrome, *ABCG2* mutations

## Abstract

**Background**: The angiotensin-converting enzyme (ACE) metabolizes a number of important peptides participating in blood pressure regulation and vascular remodeling. Elevated blood ACE is a marker for granulomatous diseases and elevated ACE expression in tissues is associated with increased risk of cardiovascular diseases. **Objective and Methodology:** We applied a novel approach —ACE phenotyping—to find a reason for conformationally impaired ACE in the blood of one particular donor. Similar conformationally altered ACEs were detected previously in 2–4% of the healthy population and in up to 20% of patients with uremia, and were characterized by significant increase in the rate of angiotensin I hydrolysis. **Principal findings:** This donor has (1) significantly increased level of endogenous ACE inhibitor in plasma with MW less than 1000; (2) increased activity toward angiotensin I; (3) M71V mutation in *ABCG2* (membrane transporter for more than 200 compounds, including bilirubin). We hypothesize that this patient may also have the decreased level of free bilirubin in plasma, which normally binds to the N domain of ACE. Analysis of the local conformation of ACE in plasma of patients with Gilbert and Crigler-Najjar syndromes allowed us to speculate that binding of mAbs 1G12 and 6A12 to plasma ACE could be a natural sensor for estimation of free bilirubin level in plasma. Totally, 235 human plasma/sera samples were screened for conformational changes in soluble ACE. **Conclusions/Significance:** ACE phenotyping of plasma samples allows us to identify individuals with conformationally altered ACE. This type of screening has clinical significance because this conformationally altered ACE could not only result in the enhancement of the level of angiotensin II but could also serve as an indicator of free bilirubin levels.

## 1. Introduction

Angiotensin I-converting enzyme (ACE, CD143) is a Zn^2+^ carboxydipeptidase, which plays key roles in the regulation of blood pressure and in the development of vascular pathology. ACE is constitutively expressed on the endothelial cell surface, absorptive epithelial, and neuroepithelial cells and cells of the immune system (macrophages, dendritic cells), as reviewed in [1,2]. Blood ACE likely originates from endothelial cells [3], primarily lung capillary endothelium [4], by proteolytic cleavage [5,6,7]. In healthy individuals, blood ACE levels are very stable over their lifetime [8], whereas in granulomatous diseases (e.g., sarcoidosis and Gaucher’s disease), blood ACE activity is significantly increased [9,10,11].

Due to the increased frequency of sarcoidosis [12], we can expect that correct and quantitative determination of ACE in the blood becomes increasingly necessary. Additionally, changes in mentality in clinical medicine toward personalized/precision medicine [13,14,15] aroused the need for more accurate determination of ACE levels, as well as ACE status (ACE phenotype) [16,17,18,19,20,21].

We established a novel approach, blood ACE phenotyping, for the purpose of full characterization of ACE in plasma or serum [17,18,19,20,21]. The kinetic and conformational aspects of ACE phenotyping allowed identification of patients with conformationally changed ACE in the blood of patients with uremia (and in a small part of the blood samples from healthy donors). This kind of ACE was characterized by an increased activity toward angiotensin I [17]. Potential clinical relevance of this finding is that the presence of such ACE levels in the blood of patients can contribute to ACE inhibitor resistance in non-responders and lead to continuous local increase in angiotensin II formation. Moreover, such ACEs could be considered to be an indicator of a risk of the End Stage Kidney Disease (ESKD), which is especially important for African Americans [22].

During routine testing of plasma from different categories of donors and patients, we found an apparently healthy donor, donating his blood for transfusion procedures, who seemed to possess conformationally changed ACE. As we had access to a large volume of plasma from this donor (plasma was expired for transfusion) we had a possibility to study the putative reasons for these changes in his ACE conformation.

We did not find ACE mutation to be the reason for these conformational changes in ACE in this donor, but we found that the changes in ACE conformation in this given patient could be explained by the presence of some plasma components, or at least increased level of endogenous ACE inhibitors. Moreover, we came to conclusion that the results of really precise determination of ACE status in the blood of any given patient are associated with the bilirubin status of this patient, because likely the level of free bilirubin in this patient not only determines an apparent conformation of ACE surface topography but also influence the rate of ACE shedding, i.e., blood ACE level. We hypothesize that the parameter we introduced, the ratios of the binding of two monoclonal antibodies (mAbs) 1G12/9B9 and 6A12/9B9, reflects the level of free bilirubin, which could be especially useful in perinatology for the detection of patients with high levels of free bilirubin, i.e., patients with high risk of toxic brain injury [23].

## 2. Materials and Methods

***Chemicals.*** ACE substrates, benzyloxycarbonyl-L-phenylalanyl-L-histidyl-L-leucine (Z-Phe-His-Leu) and hippuryl-L-histidyl-L-leucine (Hip-His-Leu) were purchased from Bachem Bioscience Inc. (King of Prussia, PA, USA) and Sigma (St. Louis, MO, USA). Other reagents (unless otherwise indicated) were obtained from Sigma (St. Louis, MO, USA).

***Antibodies.*** Antibodies used in this study include a set of 17 (mAbs) to human ACE, recognizing native conformation of the N and C domains of human ACE [24,25].

***Study participants.*** The study was approved by the Ethics Committee of the Medical Center of Moscow University (protocol # 9, 26 November 2018). All corresponding procedures were carried out in accordance with institutional guidelines and the Code of Ethics of the World Medical Association (Declaration of Helsinki). All patients provided written informed consent to have serum and citrated plasma for ACE characterization.

***ACE activity assay.*** ACE activity in serum, plasma or purified lung ACE preparations was measured using a fluorimetric assay with two ACE substrates, 2 mM Z-Phe-His-Leu or 5 mM Hip-His-Leu [26,27]. Briefly, 20 µL aliquots of serum or plasma (diluted 1/5 in PBS) or aliquots of purified lung ACE preparations with corresponding specific ACE activity, were added to 100 µL of ACE substrate and incubated for the appropriate time at 37 °C. The His-Leu product was quantified fluorometrically. ACE activity in individual patients was expressed as % from pooled citrated plasma (control) collected from plasma of healthy donors and purchased from Interstate Blood Bank, Inc. (Memphis, TN). Before pooling, each plasma sample was preliminary tested for the presence of ACE inhibitors or conformationally changed ACEs, as described in this study. ACE activity in serum/plasma was also determined with 0.3 mM angiotensin I as a substrate in PBS, pH 7.5, also using fluorimetric assay as described above.

Calculation of ZPHL/HHL ratio [27] was performed by dividing fluorescence of the sample with ZPHL to that with HHL. Human lung ACE was purified using lisinopril-affinity chromatography exactly as described before [28].

***Immunological characterization of the blood ACE.*** Microtiter (96-well) plates (Corning, Corning, NY) were coated with anti-ACE mAbs via goat anti-mouse IgG (Pierce, Rockford, IL) bridge and incubated with plasma/serum/lung ACE samples. After washing of unbound ACE, plate-bound ACE activity was measured by adding a substrate for ACE (Z-Phe-His-Leu) directly into wells [26]. The level of ACE immunoreactive protein, using strong mAb 9B9, was quantified as described previously [26]. Conformational fingerprinting of blood ACE with mAbs to ACE was performed and presented as described previously [19,24].

***Bilirubin determination.*** Plasma bilirubin levels were measured using AU480 analyzer (Beckman Coulter, USA).

***Sequencing (Sanger) and genotyping.*** Genomic DNA was obtained from the whole blood of donor 2D (and his sister) by QIAamp DNA Mini Kit (Qiagen, Valencia, CA, USA), and six exons of ACE gene (*ACE)*, 7–11th and 13th, were amplified and sequenced as in [29]. Two exons of the lysozyme gene (*LYZL1*), 2th and 4th, coding most of the amyloidogenic mutations [30], were amplified and sequenced using primers, kindly provided by Dr. T. Prokaeva (Boston University, Boston, MA). Genotyping of *UGT1A1* gene for polymorphism of TATAA repeats (UGT1A1 * 28) was performed as in [31].

***Next-Generation sequencing of exomes.*** The sequencing of 6000 + clinically relevant genes in the proband and his sister was performed by Genotek Ltd. (Moscow, Russia), after Ethics committee approved the study (07/2018). The proband and his sister gave written informed consent for studies and publication of their clinical information, images, and sequencing data. DNA libraries were constructed using the NEBNext Ultra DNA Library Prep Kit for Illumina (New England Biolabs, Ipswich, MA, USA) with adapters for sequencing on Illumina platform according to manufacturer’s protocol. For target enrichment, we used SureSelect XT2 (Agilent Technologies, Santa Clara, CA, USA). Enriched samples were sequenced using an Illumina HiSeq 2500 system (Illumina, San Diego, CA, USA) in paired-end mode (100 bp reads) and analyzed as described exactly in [32].

***Modeling of bilirubin binding to ACE.*** Coordinates of X-ray model of human ACE (PDB: 3NXQ, [33]) were downloaded from the PDB. All molecular modeling studies were performed in Molecular Operating Environment [34]. Two residues of N-acetyl-glucosamine were attached to Asn289 similar to that found for Asn45 in 3NXQ. The proteins were subjected to the “structure preparation” procedure. Hydrogen atoms were added using the Protonate 3D algorithm. The energy of the resulting structure was minimized using AMBER14EHT force-field implemented in MOE [35,36]. The proteins were minimized until the root mean square (RMS) gradient was less than 0.001 kcal/mol/Å^2^. Bilirubin was assigned MMFF94x charges and minimized using the MMFF94x force-field until the RMS gradient was less than 0.001 kcal/mol/Å^2^. The MOE docking module “Dock” was used for docking/scoring using the default parameters and settings. The approximate location of the docking site was assigned based on the outcomes of the analysis with antibodies [37,38]. Docking was performed using the “induced fit” algorithm, “Triangle Matcher” for placement, London dG for scoring of the binding poses after placement, and GBVI/WSA dG [34] for rescoring of the resulting poses. A total of 10 poses were stored after the refinement step. The docking poses that did not meet antibodies epitope mapping [37,38] were discarded, and the top remaining poses were considered for further analysis. The molecular surface colored to electrostatic potential was generated for the resulting structure except Asn45, Asn289, and Asn416 which were colored in green. All the post-translational modifications with sugars were rendered with “space fill” and colored cyan. In addition, the carboxyl groups in the top pose of bilirubin were glucuronidated, and the resulting structure was co-minimized with ACE using the same procedure as above.

***Statistical analysis.*** All experiments were conducted independently in duplicate or triplicate, the results were expressed as mean value ± standard deviation, SD. Statistica for Windows (version 10.0, Stat.Soft. Inc., Tulsa, OK, USA) was used for statistical analysis. Significance was analyzed by Mann–Whitney test with *p* ≤ 0.05 considered statistically significant and *p* ≤ 0.01 considered highly statistically significant.

## 3. Results and Discussion

**Blood ACE phenotyping and identification of patient with conformationally changed ACE.** Previously, we developed a new approach to characterize blood ACE in individual patients-blood ACE phenotyping [17,18,19,20,21]. This approach includes not just determination of ACE activity (with two substrates, ZPHL and HHL), but also determination of a novel kinetic parameter, the ratio of the rates of the hydrolysis of these two substrates (ZPHL/HHL ratio), which is able to control the native state of N and C domains of ACE active centers and to reveal the presence of ACE inhibitors [17,18,19,20,21,27]. The third parameter is the concentration of ACE immunoreactive protein [24], and, finally, the fourth and most sensitive approach is conformational fingerprinting of ACE using anti-ACE mAbs showing subtle conformational changes in ACE surface topography [17,18,19,20,21,24,39].

**Figure 1:**
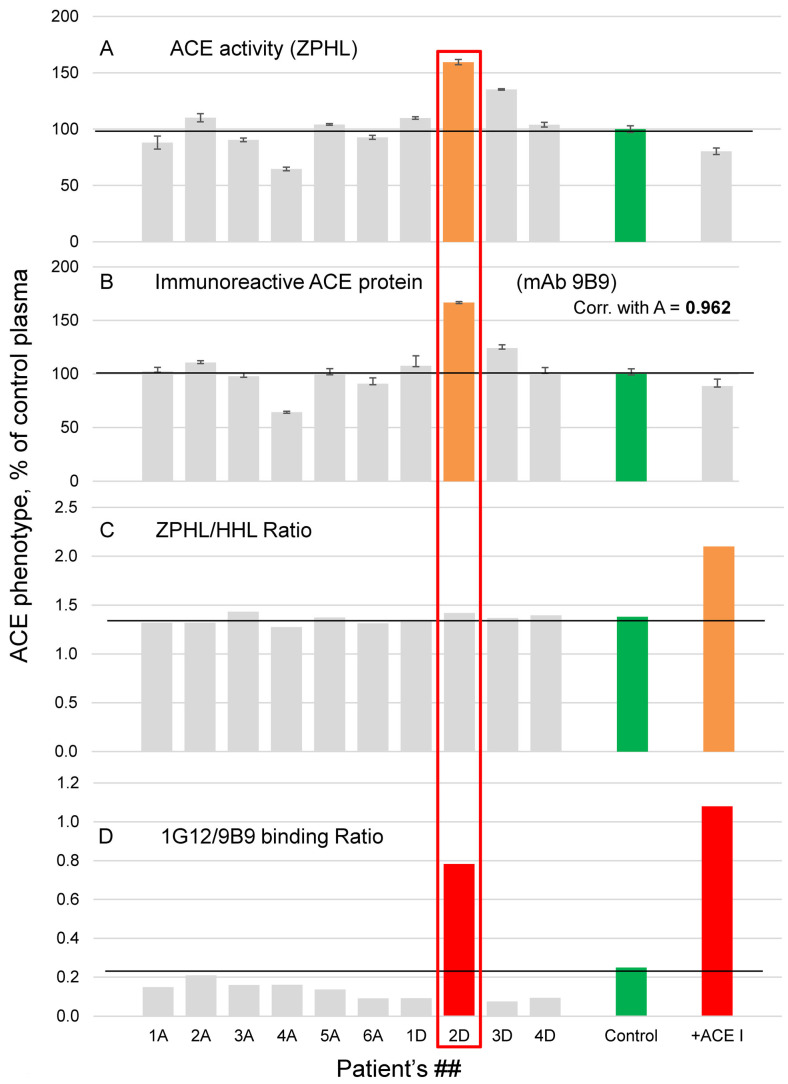
**ACE phenotyping in citrated plasma samples.** (**A**) ACE activity was measured by a spectrofluorometric assay with ZPHL (2 mM as a substrate) (**B**) The immunoreactive ACE protein was quantified by precipitation of ACE activity from plasma samples by mAb 9B9. (**C**) Ratio of the rates of the hydrolysis of two substrates, 2 mM ZPHL and 5 mM HHL (ZPHL/HHL ratio) (**D**) Ratio of ACE activity precipitation from plasma by mAbs 1G12 and 9B9 (1G12/9B9 ratio). Data were expressed as % of parameters of ACE phenotype from corresponding values for control pooled plasma samples from healthy controls (green bars). The same pooled plasma with 5 nM of ACE inhibitor enalaprilat was used as a positive control for the putative presence of ACE inhibitor in plasma samples. Bars highlighted with brown and red-samples with values of ACE parameters higher than 150% and 200% of controls. Bars for patient 2D are red boxed.

ACE phenotyping was performed on 10 citrated plasma samples from six patients obtained after therapeutic apheresis (marked as ##A) and four healthy donors (marked as ##D), for whom the plasma for transfusion was already expired. The results are presented in Figure 1 in comparison with corresponding results for previously obtained control pooled serum from 83 healthy donors. Quantification of ACE activity (with ZPHL as a substrate, Figure 1A) and the levels of ACE immunoreactive protein determined with strong mAb 9B9 (Figure 1B) demonstrated excellent correlation (R = 0.962).

We already performed ACE phenotyping in 300 apparently healthy individuals [21] and found that standard deviation (SD) from mean of ACE activity or level of ACE immunoreactive protein (with mAb 9B9) for the population was about 25% for both methods. It means that normal blood ACE values (for 95% of population) are within range of mean ± 2SD (50–150% of the mean), i.e., inter-individual variations in ACE level are significant, at least three-fold, which confirmed previous estimations [8,26]. However, one sample from 10 plasmas phenotyped in this study (from donor 2D) had ACE activity and immunoreactive ACE protein level more than 150% from the mean value (brown colored in Figure 1A,B). We also determined the ratio of the rates of the hydrolysis of two substrates, ZPHL/HHL ratio, which elevated values serves as an indicator of the presence of commercial ACE inhibitors [27], but did not find any significant elevation of ZPHL/HHL ratio (Figure 1C), indicating that all these 10 plasmas do not contain exogenous (commercial) ACE inhibitors [19,27].

The amount of ACE immunoreactive protein (Figure 1B) was estimated using the strongest mAb to ACE, clone 9B9 [38,40,41]. A great advantage of this approach is a possibility to measure ACE levels in plasma taken with EDTA or in plasma containing ACE inhibitors, because EDTA or ACE inhibitors are washed out during washing step with distilled water with Tween-20 while ACE is still bound to this mAb [19,26]. However, precipitation of ACE activity from tested plasmas was also performed with mAb 1G12, which binding to blood ACE is extremely sensitive to the presence of ACE inhibitors in the blood [17,18,19,20,21,37]. Patient 2D demonstrated dramatically increased 1G12/9B9 ratio (Figure 1D), as if this patient had ACE inhibitors in his blood (for comparison, see an effect of 5 nM ACE inhibitor enalaprilat on ACE activity precipitation from control plasma, red bars in Figure 1D) but without increase in ZPHL/HHL ratio (Figure 1C). On the base of these results, we formed a pool of citrated plasma from three plasma samples from healthy donors (not including plasma from patient 2D) which was used as a control for further experiments.

Previously, we already found several persons (3 from tested 48, i.e., approximately 6%) among healthy donors, which plasma ACE demonstrated dramatic increase in 1G12/9B9 binding ratio without concomitant increase in ZPHL/HHL ratio [17]. The proportion of patients with such conformationally changed ACE significantly increased (at least up to at least 20%) among patients with uremia [17]. Pathophysiological effects of conformationally changed ACE from such patients were the following: (1) elevated rate of the hydrolysis of angiotensin I (AI) [17], which could theoretically increase the local concentrations of AII, which in turn, has numerous deleterious cardiovascular and inflammatory effects [42,43]; (2) decreased efficacy of ACE inhibitors toward these ACEs; (3) elevated blood pressure [17]. Therefore, we could not exclude the possibility that the detection of patients with such conformationally changed ACE could be clinically relevant as a screening of patients with high risk factor for ESRD (End Stage Renal Disease). As the volume of plasma from donor 2D was rather big, it gave us an opportunity to try to find the biochemical reasons for such conformational changes in blood ACE (including possible heritability of such phenotype). It is noteworthy that the ACE phenotype in donor 2D’s blood (i.e., ACE activity, the level of immunoreactive ACE protein, and enhanced 1G12/9B9 ratio) was the same at first and second blood donations which were held at an annual interval, indicating that this phenotype is not accidental but rather an intrinsic characteristic of donor 2D.

### 3.1. Characterization of ACE in Donor 2D (with Conformationally Changed ACE)

When donor 2D plasma and control plasma were equilibrated by ACE activity with ZPHL, we found that ACE in donor 2D plasma appeared to be twice as active with angiotensin I as a substrate (205 ± 25, *p* < 0.05). Thus, we confirmed our previous finding [17] that conformationally changed ACE with increased 1G12/9B9 ratio can possess enhanced ACE activity toward this substrate (which undoubtedly is of clinical importance).

Efficacy of the inhibition of plasma ACE activity with HHL or ZPHL as substrates by common ACE inhibitor enalaprilat was reproducibly lower for plasma 2D compared to control plasma, but the difference was quite small, 5–10% (Figure S1A–C).

The effect of the presence of enalaprilat on several mAbs binding is shown in Figure S1D–F. While enalaprilat did not affect the binding of mAbs 9B9 to both control ACE and ACE from donor 2D (Figure S1D) and rather equally diminished the binding of mAb 4E3 (Figure S1E), the effect of the inhibitor on mAb 1G12 binding to control and donor 2D ACEs was strikingly different. The presence of enalaprilat dramatically increased binding of mAb 1G12 to blood ACE from control plasma, whereas it almost did not affect the binding of this mAb to blood ACE from donor 2D (Figure S1F) which is already high (Figure 1D). Previously, we found that bilirubin binds to ACE exactly in the region for epitopes for mAbs 1G12/6A12 and binding of ACE inhibitors induces dissociation of bilirubin from ACE, which leads to dramatic increase in 1G12/6A12 binding. Moreover, we demonstrated that mutation in ACE (R532W) abolished bilirubin binding to ACE and caused significant increase in ACE shedding and, therefore, increase in ACE levels in the blood [29]. Thus, increased level of ACE in the 2D plasma (Figure 1A,B), high 1G12/9B9 ratio (Figure 1D) and the absence of the effect of enalaprilat on 1G12 binding to ACE from 2D plasma (Figure S1F) indicates that donor 2D could have a mutation in ACE. This mutation could be similar, but not identical to R532W, because increase in blood ACE level in donor 2D was about 160%, in contrast to 450% for patient with mutation R532W [29]. Alternatively, there could be some non-canonical bilirubin in patient 2D, which binds less to ACE, i.e., more conjugated, or optical stereoisomer [44].

**Figure 2:**
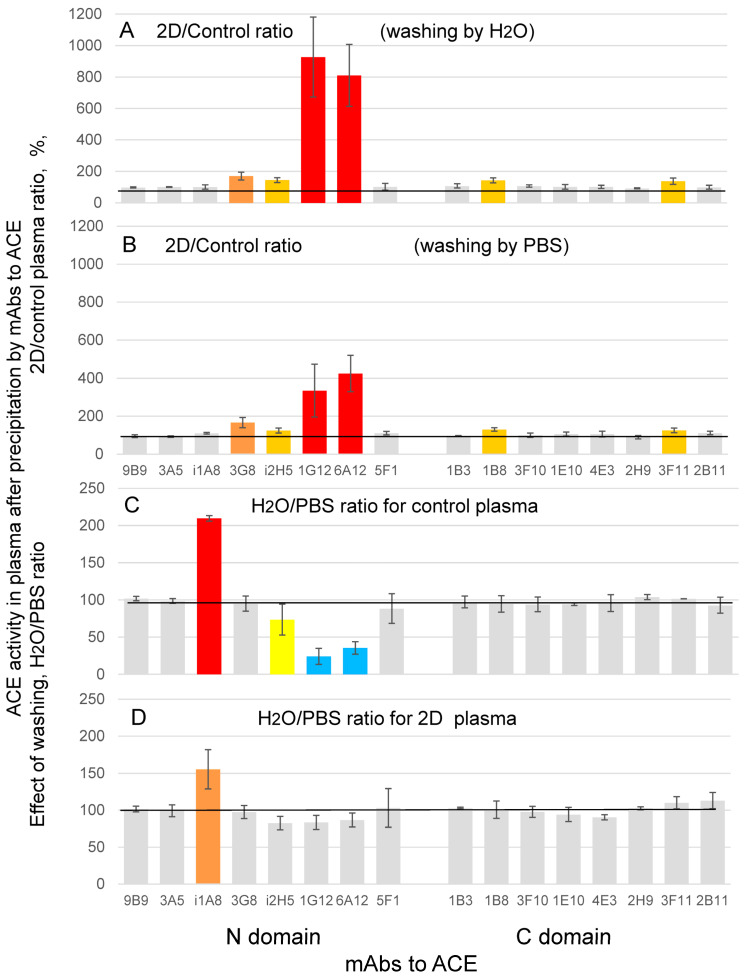
**Conformational fingerprinting of blood ACE (using mAbs to ACE).** (**A**–**D**) ACE activity was precipitated from plasma of donor 2D (boxed in Figure 1) and control plasma (diluted 1/5 with PBS) with 16 mAbs to different epitopes on the N and C domains of human ACE. (**A**,**B**) Data were expressed as a % of ACE activity from plasma of donor D precipitated by different mAbs from that for control plasma. Plates were washed from unbound ACE with distilled water (**A**) or with PBS (**B**). (**C**,**D**) Data are expressed as a % of ACE activity from control plasma (**C**) or plasma from donor 2D (**D**) with washing by water from that with washing by PBS. Orange bars-increase of ACE precipitation more than 20%, brown bars-more than 50%, red-more than 2-fold. Yellow bars-decrease of ACE precipitation more than 20%, blue bars-more than 50%.

We compared conformational fingerprint of ACE from donor 2D plasma with control plasma (Figure 2A) in order to detect the regions of possible local changes in ACE conformation in the blood of patient 2D, and to find the region of possible ACE mutation. The results for each mAb are shown as the ratio of the effectiveness of the binding of this particular mAb with ACE from 2D plasma to that with control ACE. Dramatically increased binding of two mAbs, 1G12 and 6A12, to ACE from 2D plasma closely resembles an effect of plasma dilution, filtration, dialysis, or addition of enalaprilat on mAbs binding to control plasma ACE (Figure 2C,D and Figures S12 and S13 in [29]). The similarity of Figure 2A with these figures in the cited paper allowed us to suggest that low molecular weight (LMW) blood components, which binds to ACE in normal plasma and dissociate from ACE as a result of dilution, filtration, or dialysis (or action of common ACE inhibitors inducing this dissociation) may not be able to bind to ACE in 2D plasma. It was shown [29] that this LMW blood component is bilirubin, but the possibility exists that there could be other compounds in the blood able to bind to ACE. Thus, the results of conformational fingerprinting of ACE could indicate that conformational change in ACE surface topography observed in donor 2D (Figure 2A) could be caused both by changes in ACE structure in this donor due to mutations, post-translational modifications, or changes in blood components that bind (or not bind) to ACE.

The results presented in Figure 2A as well as the experiments in Figure 2C,D and Figures S12 and S13 in [29] were obtained by washing the plates with distilled water containing 0.05% Tween-20. Strikingly, washing with PBS/Tween-20 (Figure 2B) showed fewer differences in the binding of mAbs 1G12 and 6A12 to donor 2D and control ACEs than in the case of water/Tween-20 (Figure 2A). In control plasma, washing with water/Tween-20 left a lower proportion of ACE bound to mAbs 1G12, 6A12 and, to a lesser extent, mAb i2H5 than washing with PBS/Tween-20 as evidenced by lower ACE activity (Figure 2C). A similar difference after washing with PBS or distilled water was observed with ACE purified from lung, heart, and seminal fluid (not shown). The effect appears to be focused on the same area of ACE protein as the epitopes for these very mAbs are overlapping [37,45]. Charged amino acid residues were estimated to account for 44% of the total amount of amino acid residues in the epitope area for mAb 1G12, and hydrophobic residues represent approximately 25%. For mAb 6A12, charged amino acid residues constitute 51% of amino acid residues in its epitope area, whereas hydrophobic residues account for 16% [37]. Since water/Tween-20 solution has lower ionic strength compared to that of PBS/Tween-20, it is expected to be more effective at disrupting hydrophobic interactions. Overall, these findings suggest that disruption of the hydrophobic interactions is more critical for 1G12, 6A12 and, to a lesser extent, mAb i2H5, than for other mAbs and their corresponding epitopes. The opposite is true for mAb i1A8, which showed higher remaining ACE activity after washing with water/Tween-20 compared to washing with PBS/Tween-20 (Figure 2C). Regardless of the exact reasons for this phenomenon, we stopped using distilled water for washing unbound ACE off the plate (as we did before) because very low ACE activity still bound to mAbs 1G12 and 6A12 (and, therefore, enhanced determination errors) and started using only PBS.

Unlike the control, 2D plasma showed no buffer-dependent differences in the binding of these mAbs to ACE (Figure 2D). Many factors, including mutations, post-translational modifications (PTMs), and binding of endogenous ligands, may make this region on 2D ACE surface insensitive to changes in ionic strength. Since only a few mAbs displayed the buffer-dependent binding to ACE and binding of i1A8 remained to be buffer-dependent in 2D plasma (Figure 2D), the phenomenon responsible for switching between buffer-dependent and independent states of ACE is likely to be located on a relatively small portion of the ACE protein surface.

We tested the effects of serial dilutions of control and 2D plasma and found that while the relative binding of mAbs 1G12 and 6A12 (normalized to binding of mAb 9B9) to control ACE remarkably increased upon dilution, twice more for mAb 1G12 than for 6A12 (Figure S2B,C), the binding of mAb 1G12 to ACE from 2D plasma did not depend on the dilution and relative binding of 6A12 even slightly decreased (Figure S2B,C). This result may indicate on ACE mutation/PTM in patient 2D or (alternatively) on the significant lack (or even absence) of blood components able to bind to ACE in 2D plasma, but present in the control plasma or in favor of putative endogenous ACE inhibitor/effector in 2D blood of different nature than in control plasma, which is tightly bound to ACE and does not dissociate upon dilution.

**Figure 3:**
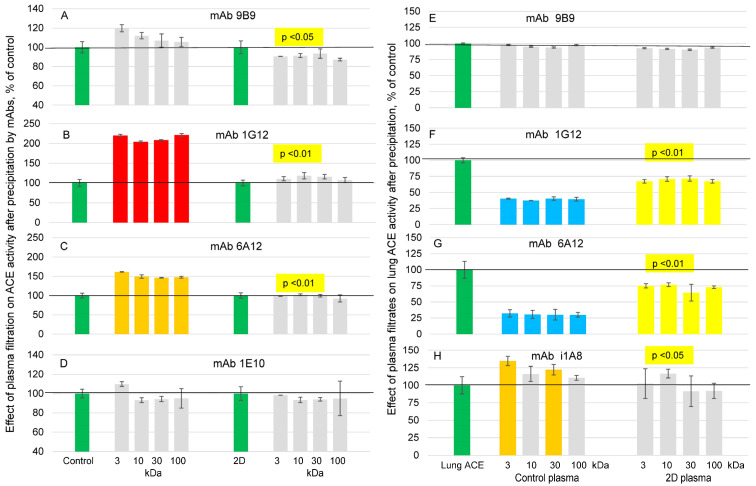
**Effect of plasma filtration and filtrates on mAbs binding to ACEs.** Plasma samples from donor 2D and control plasma (both 2 mL) were filtered by centrifugation through filters with 3, 10, 30, and 100 kD pores. (**A**–**D**) Plasmas were concentrated on filters 10-fold and then diluted 10-fold to return to initial volume. ACE activity precipitated from recovered plasmas by different mAbs was measured as in Figure 1 and Figure 2. Data were expressed as a % of precipitated ACE activity from that for initial non-filtrated plasmas (mean ± SD of triplicates) by each mAb. (**E**–**H**) Filtrates (at 90% concentration) were added to purified lung ACE (final ACE activity about 10 mU/mL). ACE activity precipitation by different mAbs was measured as in Figure 1 and Figure 2. Data were expressed as % (mean ± SD) of precipitated ACE activity in the presence of filtrates from that for controls (PBS instead of filtrates—green bars).

We tested the effect of 2D and control plasma filtration through filters of different pore size 3–100 kDa on four mAbs binding to plasma ACE. The binding of mAbs 1G12 and 6A12 to control ACE (in contrast to mAb 9B9 to the N domain and mAb 1E10 to the C domain) significantly increased after filtration (Figure 3A–D) in accordance with previous results [29] that filtration helps remove LMW ACE effector, likely bilirubin. However, filtration of 2D plasma did not increase neither 1G12 nor 6A12 binding (Figure 3C,D) which was already significantly elevated (Figure 1 and Figure 2) in comparison to control ACE, indicating that possibly bilirubin in the blood of donor 2D less binds to ACE due to some peculiarities of this ACE or decreased concentration of bilirubin (or its modifications) able to bind to this ACE. or there are other blood components in the blood of this donor that also bind to this region on N domain of ACE.

Then, we tested an effect of the filtrates from these two plasmas on the precipitation of model ACE (purified ACE from human lung) by mAbs to ACE and found that all 3-100 kDa filtrates from control plasma similarly and much more effectively decreased mAbs 1G12 and 6A12 binding to purified ACE than filtrates from donor 2D (Figure 3E–H). The effect of filtration and filtrates on ACE precipitation by 9 tested mAbs (Figure S3) confirmed reciprocal (mirrored) effect of filtration and filtrates on mAbs 1G12 and 6A12 binding. These results indicated that local conformational changes in ACE surface topography in plasma of donor 2D could be due to at least two factors—changes in ACE structure preventing effective binding of bilirubin to the epitopes of these 2 mAbs to ACE or the lack of LMW ACE-binding components in 2D plasma.

To clarify this, we purified ACE from both plasmas by affinity chromatography (batch procedure) on Lisinopril-Sepharose (Figure S4A,B). It appeared that long (24–48 h) incubation of plasma with Lisinopril-Sepharose during a batch procedure likely influenced local conformation of ACE surface topography in the region of overlapping epitopes of mAb 1G12 and 6A12, as the binding of these two mAbs was not increased (due to elimination of bilirubin after passing of plasma through Lisinopril-Sepharose column, as in Figure 10B in [39]) but significantly decreased after purification (Figure S4A,B). The only difference between the effects of purification on ACE from 2D and control plasma was less binding of mAb 6A12 to purified ACE from 2D plasma compared to control (red box). Nevertheless, conformational fingerprint of ACE partially purified from plasma of donor 2D by affinity chromatography was almost identical to that for ACE purified from control plasma (Figure S4C), in a sharp contrast to that in whole plasmas (Figure S4D), thus ruling out genuine changes in ACE conformation in donor 2D. The purification procedure dramatically changed binding of few mAbs to purified ACEs from both plasmas, and especially dramatically increased binding of mAb 5F1 (red bars in Figure S4A,B), localized in the interface of dimerization [20,46]. This fact may indicate that purification on Lisinopril-Sepharose can decrease the extent of dimerization of ACE from plasma, unmasking the epitope for mAb 5F1.

Sequencing of five exons (from 7th to 11th) coding the surface of the N domain of ACE, where overlapping region of mAbs 1G12 and 6A12 were localized [37], did not reveal any ACE mutation (Figure S5), confirming that the amino acid replacements in ACE molecule are not responsible for an apparent changed surface topography of plasma ACE of donor 2D. Nevertheless, confirming the power of personalized/precision medicine approach, we found polymorphic variant of ACE gene in this patient, heterozygous genotype CT of rs4613 in the 13th exon, coding Pro27 in the signal peptide of testicular ACE. (File S1). Homozygous TT genotype is associated with significant decrease in testicular ACE expression on spermatozoa and lower fertilization rates [47]. As we previously found that another blood component, lysozyme, binds to ACE [29], we also sequenced 2th and 4h exons of lysozyme (where most of the amyloidogenic mutations were localized [30]) and also did not found any mutation in these two exons of lysozyme gene of donor 2D.

### 3.2. ABCG2 Mutation in Donor 2D

After the experiments described above, the changes in bilirubin metabolism in patient 2D became an alternative hypothesis for the explanation of conformational changes in its blood ACE. There are numerous genes (and their products-proteins) that participate in bilirubin synthesis and metabolism, members of UTG family, biliverdin reductases, membrane transporters, for review see [48,49,50]. Therefore, in order to find causal mutation(s) which could be responsible for an apparent conformational changes in ACE surface topography in plasma of this donor, we performed exome sequencing of 6000+ clinically relevant genes in donor 2D and her sister who had no conformational changes in her plasma ACE (File S1, Figure S5).

We did not find any mutation in ACE and lysozyme genes of donor 2D nor any loss-of-function mutation in the *UGT1A1* gene [51], the product of which, bilirubin UDP-glucuronosyltransferase 1, is the only relevant enzyme responsible for bilirubin glucoronidation [52], but found several mutations (polymorphic variants) in some members of UGT family (Supplemental Materials File S1), and, most important, found mutation (M71V, rs148475733) in membrane transporter *ABCG2*, which participates in transport of more than 200 compounds (for review see [53]) including bilirubin [54]. The same mutation was described recently [55] and was shown to lead to decreased surface expression of ABCG2, but influence of this mutation on bilirubin metabolism was not analyzed.

**Figure 4:**
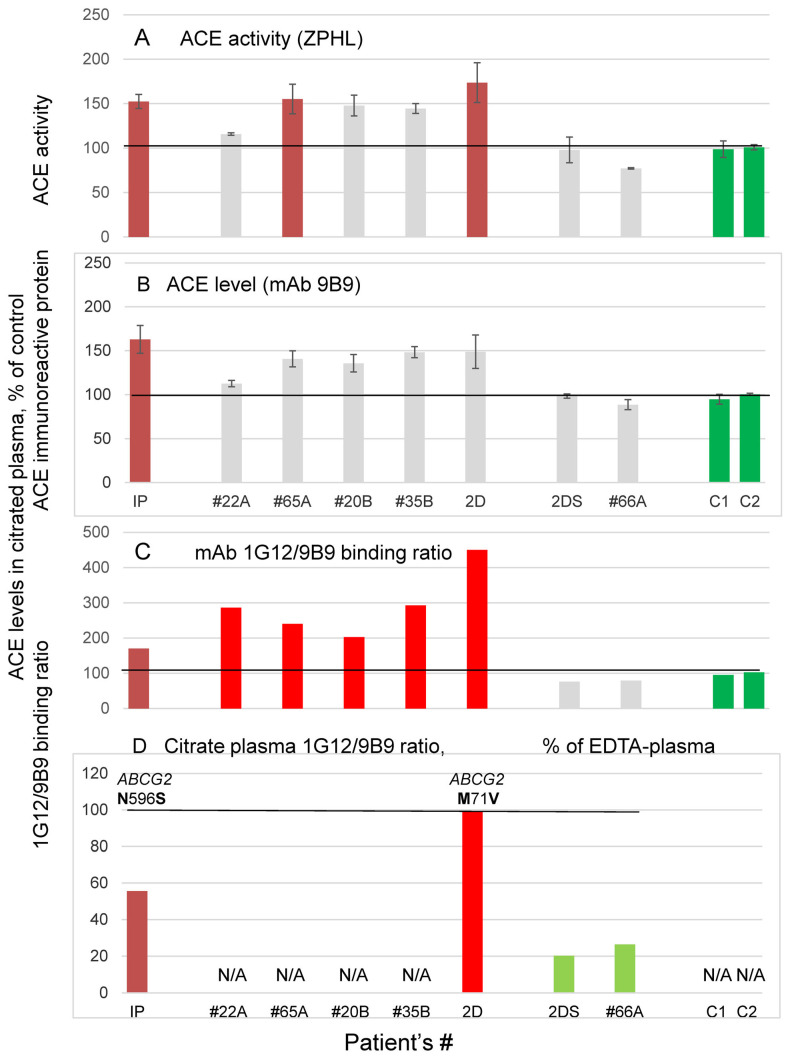
**ACE phenotyping in patients with conformationally changed ACEs**. (**A**) ACE activity; (**B**) amount of immunoreactive ACE protein determined with mAb 9B9: (**C**) and 1G12/9B9 binding ratio were quantified and presented as in Figure 1. (**D**) 1G12/9B9 binding ratio in citrated plasmas was calculated as a % from that in available EDTA plasma. As a control, we used pooled plasma samples from two different pools (1–2). Bars highlighted as in Figure 2.

Soon after that, we found plasma sample (from unrelated patient with chronic prostatitis) also with conformationally altered ACE (Index patient IP in Figure 4). Accidentally, this patient previously ordered exome sequence of 6000+ clinically relevant genes and we found that this patient has also mutation of *ABCG2*, but another one, N596S, implicated in protein trafficking and stability [53,56]. ACE phenotyping of citrated plasma from all patients with conformationally altered ACE (increased 1G12/9B9 ratio) that were found in 200 unrelated plasma samples and patients with two mutations in *ABCG2* are shown in Figure 4. Most of the patients with increased 1G12/9B9 ratio demonstrated also elevated level of blood ACE (Figure 4A,B), which is consistent with the hypothesis that these patients could have reduced level of bilirubin, which normally prevents an excessive ACE shedding and an appearance of extra ACE in the blood [29]. We expressed the 1G12/9B9 binding ratio in control citrated plasma as a percentage of this ratio in control EDTA plasma, where EDTA causes dissociation of zinc-ions from the active centers of ACE and, thus, reversibly inactivates the enzyme. We found that the epitope for 1G12 in citrated plasma of two control patients, 2DS and #66A, was unmasked by 20% in comparison to EDTA plasma (Figure 4D). The strikingly different results were obtained for donor 2D and Index patient: while in Index patient the epitope for mAb 1G12 in citrated plasma was unmasked by 50%, in donor 2D this epitope was completely unmasked in both citrate and EDTA plasma.

Thus, it is possible that some (but perhaps not all) mutations in *ABCG2* could lead to changes in bilirubin metabolism, which in turn, could influence an apparent local ACE surface conformation (1G12/9B9 binding ratio) in plasma. Therefore, we expected *ABCG2* null mutations to have more influence on bilirubin metabolism than other heterozygous mutations which we already analyzed. However, when we performed ACE phenotyping in plasma from three different *ABCG2* null mutations (Figure S6) in three patients. We did not find any effect of these null mutations on 1G12/9B9 binding ratio. The only reasonable hypothesis we could make is that the effect of tested *ABCG2* mutations (at least M71V and N596S) on bilirubin metabolism might be realized via homo- or even heterodimerization of *ABCG2* [53]. In the case of *ABCG2* null mutations, there could be no partners for homo- or heterodimerization of *ABCG2* and, as a result, no effect on bilirubin metabolism.

### 3.3. Molecular Modeling of Bilirubin Binding to ACE

**Figure 5:**
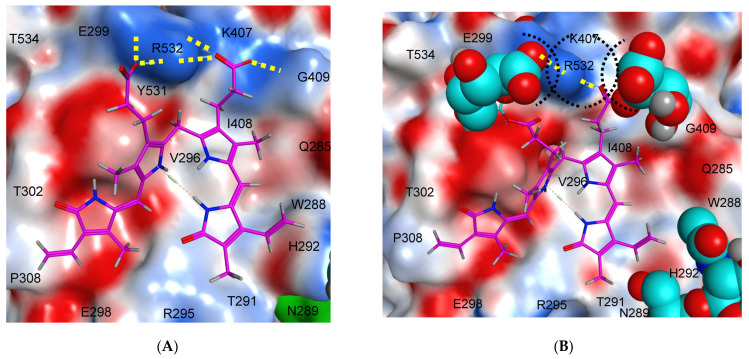
**Protein-ligand interaction between ACE and bilirubins**. N domain of human ACE (PDB: 3NXQ) with docked bilirubin (**A**) and bilirubin diglucuronide (**B**). Bilirubin scaffold is rendered by magenta. The hydrogen bonds with Arg532 are shown as yellow dotted lines. The steric interactions between the glucuronidate portions of bilirubin and Arg532 are marked by black dotted lines. The solvent accessible protein surface is mapped by electrostatic potential, red-negative, blue-positive. Gln289 is rendered as green surface. The sugar PTMs portions (Post-Translational Modifications) are rendered by cyan.

**Figure 6:**
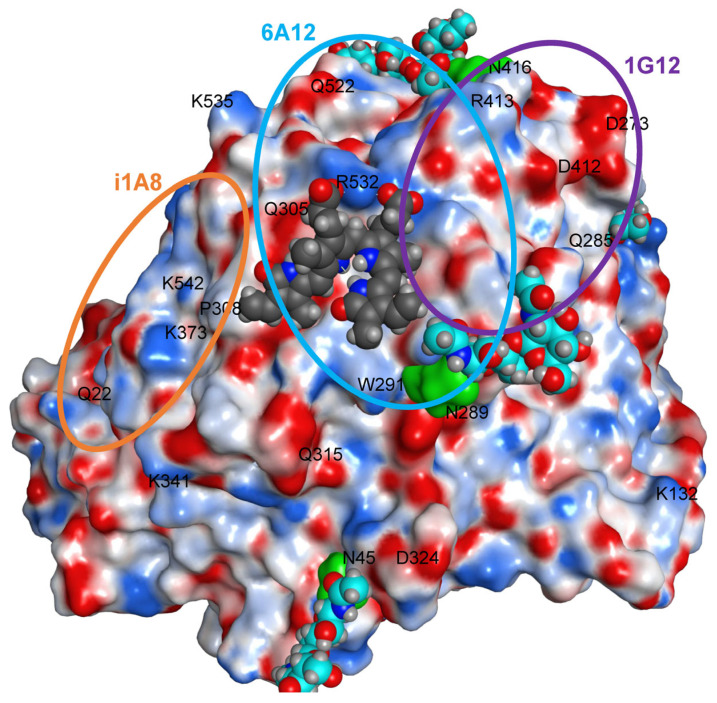
**General view of bilirubin docked to ACE.** N domain of human ACE (PDB: 3NXQ) near the epitopes for mAbs 6A12, 1G12 and i1A8. The surface of the protein is mapped by electrostatic potential, red-negative, blue-positive. Asn 45, Asn289 (Q in 3NXQ), and Asn416 are rendered by green surface. The sugar PTMs portions are rendered by cyan and bilirubin molecule -by dark gray.

To gain additional insights on the possible binding mode of bilirubin to ACE, we docked it to the putative binding site previously identified that involved R532 on the N domain of ACE [29]. The top pose of bilirubin on the human N domain of ACE (PDB: 3NXQ) is shown in Figure 5 and Figure 6. As shown in Figure 5A, bilirubin binds to a shallow hydrophobic pocket formed by the hydrophobic residues Pro308, Ile408, and Val296 and the hydrophobic portions of the side-chains of Lys407, Tyr531, Glu299, Thr302, Glu298, Arg295, Thr291, His292, and Trp299. The two negatively charged carboxyl groups of bilirubin participate in bidentate ionic interactions with positively charged Arg532 (yellow dotted lines). In addition, one of the carboxyl groups of bilirubin forms hydrogen bond with C=O of Gly409, making the resulting binding even stronger.

Mutation R532W is expected to disrupt these strong ionic interactions, resulting in a weakening bilirubin-ACE interaction as demonstrated in [29]. The docked molecule of bilirubin is only c.a. 10 Å away from Asn289, suggesting that glycan at potential glycosylation site Asn289 may potentially interfere with bilirubin binding to ACE (note proximity of the glycan rendered by cyan to bilirubin rendered by magenta (Figure 5A)).

Next, we analyzed how glucuronidation of bilirubin may impact its binding to ACE (Figure 5B). Glucuronidation of bilirubin leads to an increase in bulk of the resulting adduct in the region next to the newly formed ester bond between bilirubin and glucuronide portions. Moreover, in the glucuronidated bilirubin, the position of the negatively charged carboxyl groups are extended relatively to the heterocyclic core of bilirubin. Altogether, these changes in the structure of the glucuronidated bilirubin result in c.a. 2 Å shift in its position toward modeled Asn289 and loss of at least one strong electrostatic interaction with Arg532. A preliminary modeling shows that the carboxyl groups of the two glucuronidate groups may still form bidentate interactions with Arg532, but it would also require dissociation of the bilirubin core from the only hydrophobic pocket at the intersection of the epitopes for antibodies 6A12 and 1G12 (Figure 6). It is tempting to speculate that these changes would also disrupt the binding of glucuronidated bilirubin to ACE.

A comparison of bilirubin binding site on ACE with that on PPARα (peroxisome proliferator-activated receptor-α (docked structure, Figure 2A in [57]) and on albumin-PDB:2VUE [58]) shows that in all the cases the carboxylic acid groups of bilirubin form ionic interactions with the positively charged residues of the binding site whereas the remaining part of the ligand interacts with the hydrophobic portion of the binding site. For instance, ionic interactions between Arg117 and Arg186 on albumin with carboxylic groups of bilirubin are reminiscent to the interaction between bilirubin docked to ACE and Arg532.

Magnification of bilirubin docking model shows how bilirubin in complex with ACE maybe located in both epitopes for mAbs 1G12 and 6A12 (Figure 6). This picture also helps explain an unusual effect of filtrates of control plasma (i.e., mainly bilirubin in these filtrates) on an increase of mAb i1A8 binding to purified lung ACE (Figures S3B and S4D). Likely, binding of bilirubin to the area within epitopes for mAbs 1G12 and 6A12 changes local conformation of ACE remotely (in the region of epitope for mAb i1A8 (probably bumps formed by Q305, P308 and maybe K542)).

**Figure 7:**
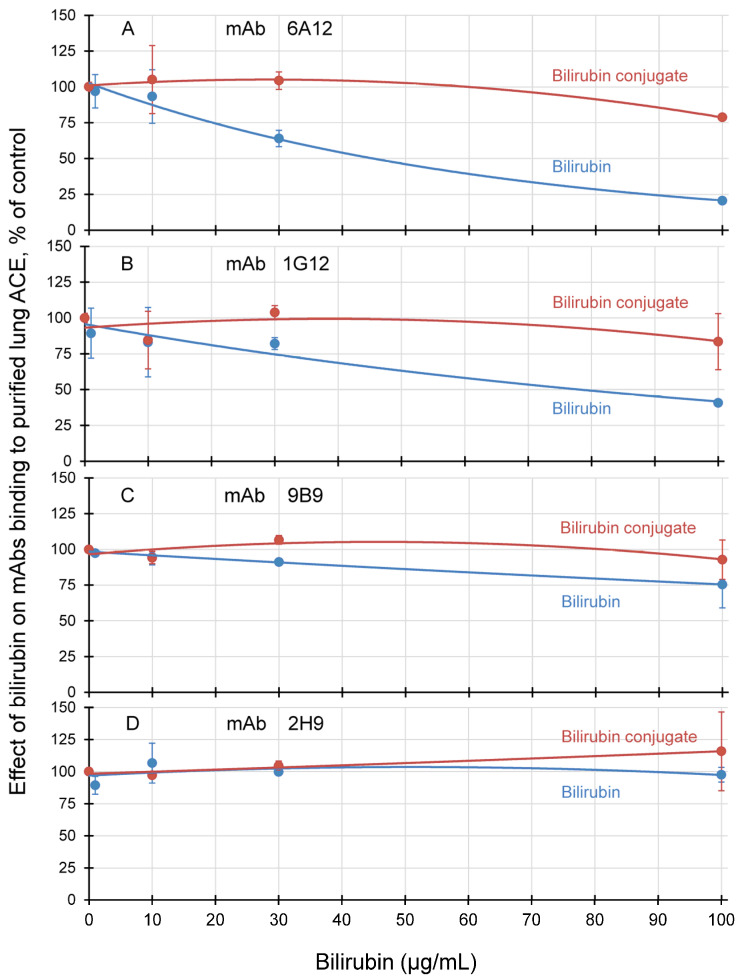
**Effect of bilirubins on mAbs binding to purified human lung ACE.** Bilirubin and bilirubin tartar conjugate in PBS were added to purified lung ACE (final ACE activity about 10 mU/mL). ACE activity precipitation by different mAbs was measured with as in Figure 1 and Figure 2. (**A**) mAb 6A12; (**B**) mAb 1G12; (**C**) mAb 9B9; (**D**) mAb 2H9. Data were expressed as % (mean ± SD) of precipitated ACE activity in the presence of bilirubins from that for controls (PBS instead of bilirubins).

We compared the effects of free and conjugated (tartar) bilirubin on the binding of mAbs to purified lung ACE (Figure 7). Whereas bilirubin did not affect the binding of mAb 2H9 to the C domain and only slightly affected the binding of mAb 9B9 to the N domain (Figure 7C,D), both bilirubin remarkably decreased the binding of mAbs 6A12 and 1G12 to the N domain of ACE (Figure 7A,B). The ability of conjugated bilirubin to decrease binding of mAbs 6A12/1G12 was the same, while free bilirubin apparently better disrupted mAb 6A12 interactions with ACE, in accordance with the above modeling and results in [29].

It should be emphasized that the effect of free bilirubin on mAb 6A12 (and 1G12) binding was much more pronounced than the effect of the same concentrations of conjugated bilirubin (Figure 7A,B) confirming the conclusions that free bilirubin better binds to ACE molecule.

Very rough estimation of the comparative efficacy of free and conjugated bilirubins (Figure 7A) toward 6A12/9B9 ratio showed that free bilirubin at least five-fold better binds to ACE than conjugated form. In normal conditions, however, the concentration of direct bilirubin is two orders higher than free unbound bilirubin. Therefore, there are much more complexes of ACE with direct bilirubin than complexes with free bilirubin. At the pathological level, however, increased or decreased concentration of free bilirubin might influence on the proportion of the complexes of ACE with both bilirubin.

Much evidence suggests that free (unbound and unconjugated) bilirubin concentration correlates more strongly with bilirubin toxicity than the total bilirubin [59]. These results justified further investigations into the clinical use of free bilirubin measurements [60]. Unfortunately, accurate measuring the free bilirubin concentration in the presence of much higher concentrations of protein-bound bilirubin is difficult [61]. However, at least two reliable methods for the measurement of free bilirubin (Bf) were established. Green fluorescent protein from eel was cloned, the fluorescence of which was significantly increased in the presence of free bilirubin [62]. In another approach, fluorescently labeled mutants of fatty acid binding proteins were used [63]. This sensor binds unconjugated bilirubin with high affinity (Kd = 16 nM) but binds conjugated bilirubin much worse, Kd > 300 nM [64]. Both methods started to be used for clinical determination of free bilirubin in human plasma [64,65]. This discrimination in free and conjugated bilirubin binding is similar to the effect of these bilirubins on ACE binding by mAbs 1G12 and 6A12 (Figure 7). Therefore, we may speculate that the 6A12/9B9 (or 1G12/9B9) ACE-binding ratio could be an additional (and natural) sensor for free bilirubin determination in the blood of patients.

### 3.4. Endogenous ACE Inhibitors in Plasma of Donor 2D

We analyzed an effect of dilution and filtration of plasma 2D and control plasma, as well as an effect of filtrates of plasmas on purified ACE, not only on mAbs binding (Figure 3 and Figure 4), but also on ACE activity (Figure S7). Filtration of 2D and normal plasmas (Figure S7D,E) shows that this procedure resulted in a remarkable increase of ACE activity in 2D plasma, but only toward the substrate HHL, which in turn, indicates the presence in 2D plasma of endogenous ACE inhibitors preferably inhibiting the C domain of the enzyme. This conclusion is supported by the findings that simple 2D plasma dilution (Figure S7C) and filtration (Figure S7F) significantly decreased ZPHL/HHL ratio for plasma ACE, while filtrates of this plasma increased ZPHL/HHL ratio for purified ACE (Figure S7F). Therefore, while the balance of endogenous ACE inhibitors in normal plasma is shifted toward inhibitors preferably inhibiting the N domain [27], the balance of ACE inhibitors in 2D plasma is shifted toward inhibitors preferably inhibiting the C domain of the enzyme. The overall number of inhibitors in 2D plasma is apparently higher than in normal plasma as the effect of 2D filtrates on the activity of purified ACE is greater (Figure S7H).

In addition, we found that ACE activity in the serum was higher than that in citrated plasma for almost all tested normal blood samples by 11.4 ± 7.7% (mean for nine samples) (Figure S8A), indicating that some ACE inhibitor(-s) binds to the blood clot. However, this difference was much more pronounced for donor 2D (+58.0 ± 4.4%), while the amount of immunoreactive ACE protein was the same in his serum and plasma (Figure S8B). Therefore, we can speculate that concentration of this endogenous ACE inhibitor(s) is five-fold higher in 2D plasma than in plasma of nine healthy volunteers. Purification of ACE from normal and 2D plasma also revealed that ACE binding with Lisinopril-Sepharose was more effective for control plasma than ACE from 2D plasma as less ACE activity appeared under washing: 18.6% ± 10.8 for control plasma versus 48.9% ± 19.5 (*p* < 0.001) for plasma 2D. This fact could be attributed to the enhanced concentration of ACE inhibitors in 2D plasma competing with Lisinopril on the matrix.

Conformational fingerprinting of ACEs in serum versus plasma for donor 2D (and his unaffected sister) indicated that the elimination of the inhibitor(s) binding by blood clot somewhat changed the efficacy of binding of mAb 6A12 to the N domain and mAbs 1E10 and 4E3 to the overlapping region of their epitopes on the C domain (Figure S8C,D).

Therefore, in addition to decrease in bilirubin binding, the changes in content and concentration of endogenous ACE inhibitors can change an apparent local surface ACE conformation in plasma.

### 3.5. Blood ACE Phenotyping in Patients with Gilbert and Crigler-Najjar Syndromes

Bilirubin is the final product of heme catabolism, mainly originating from hemoglobin after degradation of old erythrocytes. In healthy individuals, the total level of blood bilirubin is under 17 μM [66]. Bilirubin is poorly soluble in water and in the blood is mostly bound to serum albumin [50], while water soluble (or direct) bilirubin results from conjugation of the initial molecule with one or two glucuronide groups by the enzyme UDP-glucuronosyl transferase 1A1 (UGT1A1) [52]. Plasma (serum) total and direct bilirubin concentrations are common laboratory criteria of bilirubin status in many diseases, but especially in jaundiced newborns [67]. Displacement of bilirubin from albumin binding sites or mutations in *UGT1A1* gene leading to a decreased expression of UGT1A1 are the reasons for enhanced levels of free, unbound bilirubin which usually presents in very small quantities in the blood and is highly neurotoxic. The aftermath health effects could be Gilbert syndrome (with a frequency of up to 15% in the Western population) with up to 90 μM total bilirubin [31], which is considered relatively harmless, but jaundice can be triggered by different types of stress. Rather rare but much more severe case is Crigler-Najjar syndrome with ≤10% UGT1A1 activity and a total bilirubin level in a range 100–750 μM (depending on I or II Type) with a risk of brain damage in infancy and teenage years, including encephalopathy and neurological impairment [23].

**Figure 8:**
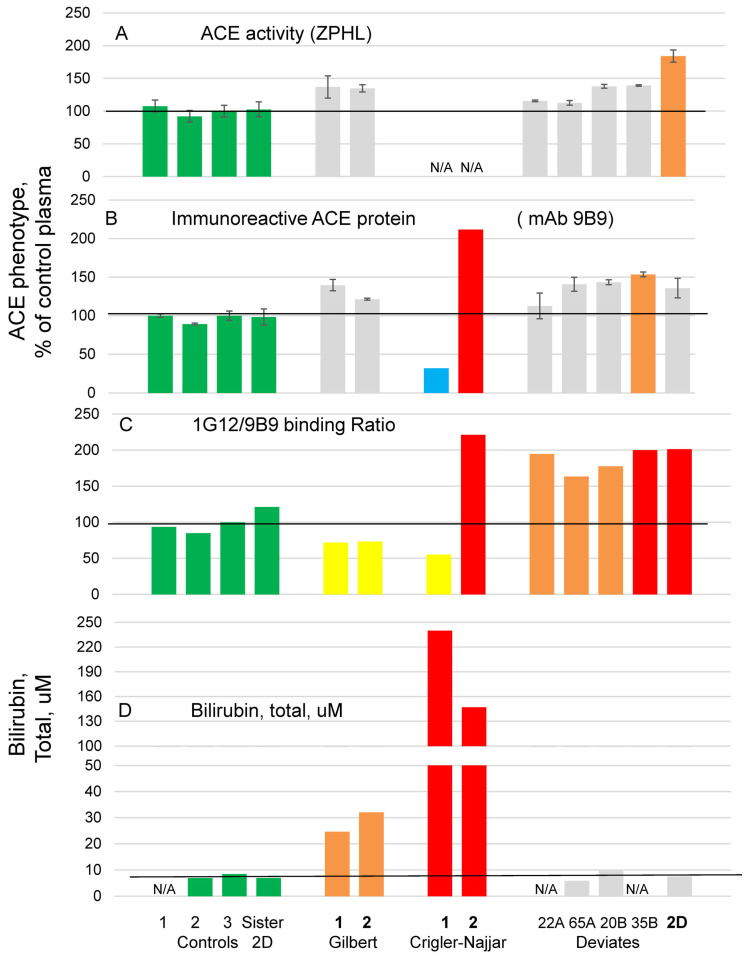
**ACE phenotyping and bilirubin status in patients with Gilbert and Crigler-Najjar syndromes.** ACE activity (**A**), amount of immunoreactive ACE protein determined with mAb 9B9 (**B**) and 1G12/9B9 binding ratio (**C**) were quantified and presented as in Figure 1. The available data on total bilirubin concentration are also presented (**D**). As controls, we used plasma samples from pools 1-2-3 and from sister of donor 2D. Several plasma samples with conformationally changed ACE, which we found in apparently healthy population, as well as plasma from donor 2D, are presented for comparison. Bars highlighted as in Figure 2.

Previously, we showed that bilirubin is able to bind to ACE and thus cause the decrease of the 1G12/9B9 binding ratio [29]. The modeling of bilirubin-ACE interaction shown in the present work demonstrates that both direct conjugated and free unconjugated bilirubins can bind to ACE, but free bilirubin is able to form the most favorable interactions with the enzyme. Thus, we performed plasma ACE phenotyping in two patients with Gilbert syndrome (from Russia) and two patients with Crigler-Najjar syndrome (Type II, from Netherlands) and compared it with four patients with conformationally changed ACE, exhibiting increased 1G12/9B9 ratio, which we found in an independent study, along with 2D plasma (Figure 8, clinical details are in File S2). As negative controls, we used three different pools of citrated plasmas from patients with native ACE according to their ZPHL/HHL and 1G12/9B9 ratios [17] and plasma from the sister of donor 2D, also with naïve ACE conformation.

ACE activity was not measured quantitatively in the two patients with Crigler-Najjar syndrome (Figure 8A) due to very high concentrations of bilirubin in these two patients, 240 and 147 μM (Figure 8D), which interfered with fluorimetric assay of ACE activity, but the amount of immunoreactive ACE protein, measured with mAb 9B9, appeared to be very different (5-fold) in these two patients (Figure 8B). Both ACE activity and the amount of immunoreactive ACE protein in plasma of patients with Gilbert syndrome with mild hyperbilirubinemia −24.6 and 32 μM (Figure 8D) were rather similar and slightly higher than in control plasmas (Figure 8A,B).

The values of 1G12/9B9 ratio were significantly decreased compared to normal values for both patients with Gilbert syndrome (Figure 8C) in accordance with earlier observation [29] that bilirubin can bind to ACE and thus decrease 1G12/9B9 ratio. We can hypothesize that these patients may have elevated levels of free bilirubin. This conclusion is supported by increased conjugated/serum total bilirubin ratio in patients with Gilbert syndrome [66].

For patients with Crigler-Najjar syndrome, however, the situation appeared to be equivocal. While ACE in the plasma of patient #1 with extremely high, total bilirubin was characterized by a low 1G12/9B9 ratio; the ACE in plasma of patient #2, also with high total bilirubin, unexpectedly, exhibited a high 1G12/9B9 ratio (Figure 8C,D). It is noteworthy that patients with Crigler-Najjar syndrome are characterized by much lower expression of UGT1A1 and higher extent of hyperbilirubinemia than Gilbert syndrome [48]. We could also expect higher amount of free bilirubin in plasma of these two patients than in normal plasma and plasma of patients with Gilbert syndrome and, therefore, even lower values of 1G12/9B9 ratio, as well as the decrease in blood ACE levels. It appeared to be true, but only for patient #1 (Figure 8B,C), while patient #2 demonstrated significantly elevated 1G12/9B9 ratio Figure 8C) and the level of ACE in plasma (Figure 8B). The primary hypothesis is that, despite dramatic hyperbilirubinemia, patient #2 has much lower levels of free bilirubin and thus this patient is not in a risk group for bilirubin-induced encephalopathy, because the concentration of free bilirubin in his plasma should not be high (and toxic). (Alternative explanation could be that patient #2 may have conformationally impaired ACE). The only way to clarify the reason for such differences in mAbs to ACE binding for these two patients with Crigler-Najiar syndrome is to measure free bilirubin by independent methods [64,65]. Unfortunately, none of these methods are available yet.

We also performed ACE phenotyping in 100 apparently healthy volunteers and calculated 1G12/9B9 ratio for those patients who had no ACE inhibitors in their blood (Figure S9). We found four patients with increased 1G12/9B9 ratio (orange bars) in accordance with previous results [17] demonstrating that apparently healthy donors could have conformationally altered ACE in their blood and, therefore, are at risk of hypertension and renal disease development. However, we also found nine patients with significantly decreased 1G12/9B9 ratio (yellow bars). Based on the results of 1G12/9B9 ratio calculation in two patients with Gilbert in Figure 8C we can hypothesize that these nine patients with 1G12/9B9 ratio less than 80% may have elevated levels of free bilirubin and, therefore, could be candidates for Gilbert syndrome. High frequency of Gilbert syndrome in Caucasians [31] fit with this hypothesis.

## 4. Conclusions

We can state that donor 2D (with conformationally impaired ACE) characterized by significantly increased activity toward angiotensin I, is also characterized by significantly increased level of endogenous ACE inhibitor (with MW less than 1000) which likely induced dissociation of bilirubin from its binding site near the epitope for mAb 6A12.

Moreover, this donor has a mutation in membrane transporter *ABCG2* participating in transport of more than 200 compounds, including bilirubin. We also demonstrated by modeling and confirmed by in vitro experiment that free bilirubin binds more effectively to its binding site on the N domain of ACE, than conjugated bilirubin.

Therefore, our data allowed us to hypothesize that we made a starting point for the indirect method that can reflect probable concentration of free bilirubin in plasma (serum). Estimation of (patho)physiologically active free bilirubin could be diagnostically extremely interesting, because an excess of free bilirubin is very toxic for the brain and clinical measurement of free bilirubin is limited to the small numbers of very big and well-equipped hospitals.

## Data Availability

All data are included in the manuscript.

## Supplementary Materials

The following supporting information can be downloaded at: https://www.mdpi.com/article/10.3390/biomedicines11020534/s1.
