# Supplementary material for: Blood ACE Phenotyping for Personalized Medicine: Revelation of Patients with Conformationally Altered ACE
Source: Biomedicines. 2023 Feb 13;11(2):534. doi: 10.3390/biomedicines11020534 (PMC9953529; doi:10.3390/biomedicines11020534)
Supplement: Supplementary file 1 [file biomedicines-11-00534-s001.zip › biomedicines-2210820-supplementary.pdf]

biomedicines

Supplementary Materials

Blood Ace Phenotyping For Personalized Medicine: Revelation Of Patients With Conformationally Altered Ace.

Sergei M. Danilov [1,2,3,*], Mark S. Jain [3], Pavel A. Petukhov [4], Olga V. Kurilova [3], Valery V. Ilinsky [5], Pavel E. Trakhtman [6], Elena L. Dadali [7], Larisa M. Samokhodskaya [3], Armais A. Kamalov [3] and Olga A. Kost [8]

1 Department of Medicine, Division of Pulmonary, Critical Care, Sleep and Allergy, University of Illinois, Chicago, IL 60607, USA
2 Department of Medicine, University of Arizona Health Sciences, Tucson, AZ 85721, USA
3 Medical Center, Lomonosov Moscow State University, 119992 Moscow, Russia
4 Department of Pharmaceutical Sciences, College of Pharmacy, University of Illinois, Chicago, IL 60612, USA
5 Genotek, Ltd., 105120 Moscow, Russia
6 Dmitry Rogachev National Medical Research Center of Pediatric Hematology, Oncology and Immunology, 117997 Moscow, Russia
7 Medico-Genetic Center, 115478 Moscow, Russia
8 Chemistry Faculty, Lomonosov Moscow State University, 119991 Moscow, Russia
* Correspondence: danilov@uic.edu

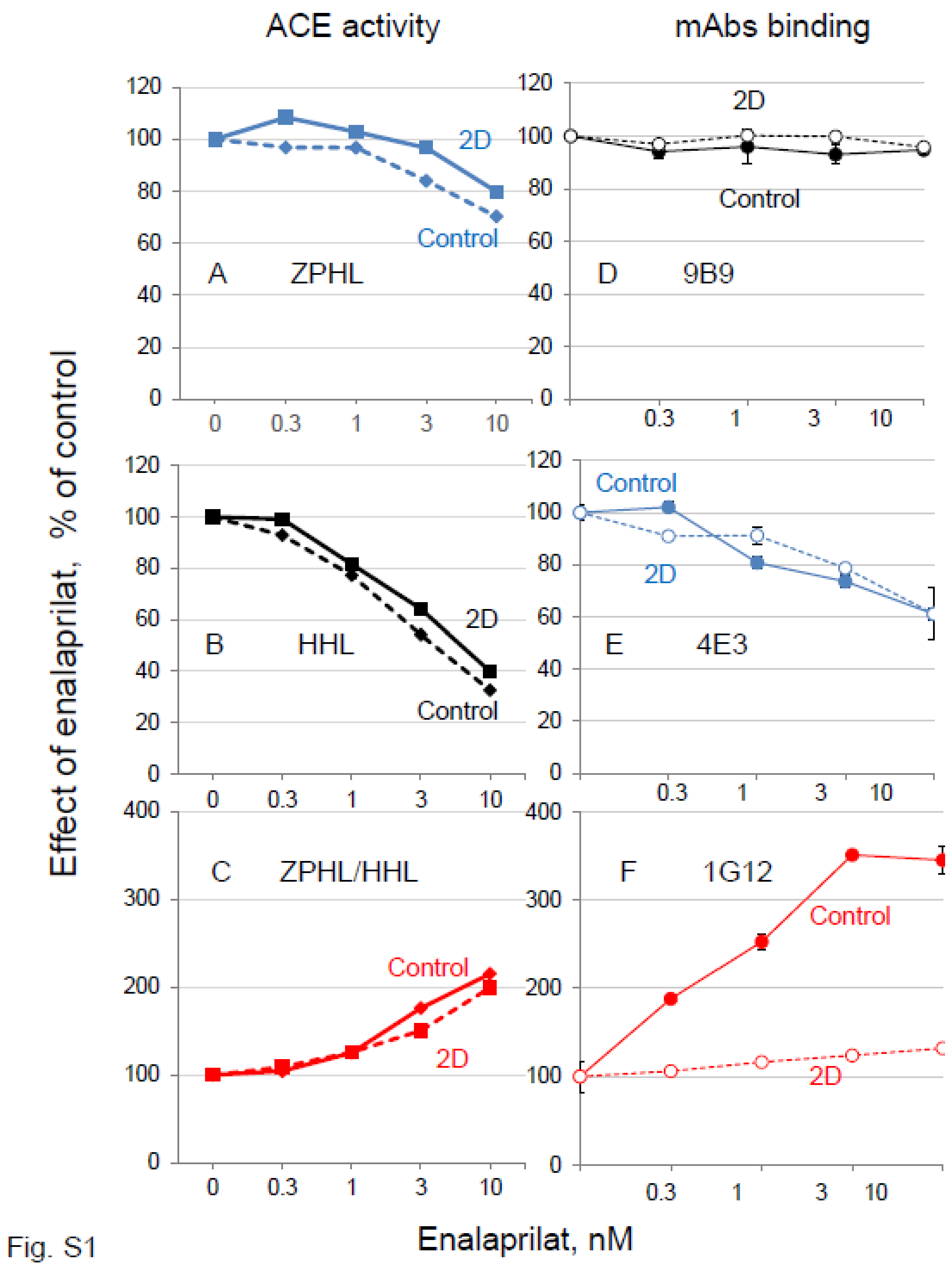

Figure S1. Effect of ACE inhibitor enalaprilat on ACE from donor 2D. Plasma from donor 2D (and control plasma), both in 1/5 dilution in PBS were incubated with indicated final concentrations of enalaprilat and then ACE activity was quantified with two substrates (**A-C**) and precipitation of ACE activity was performed with mAbs 9B9, 4E3 and 1G12 (**D-F**) as in Fig.1.

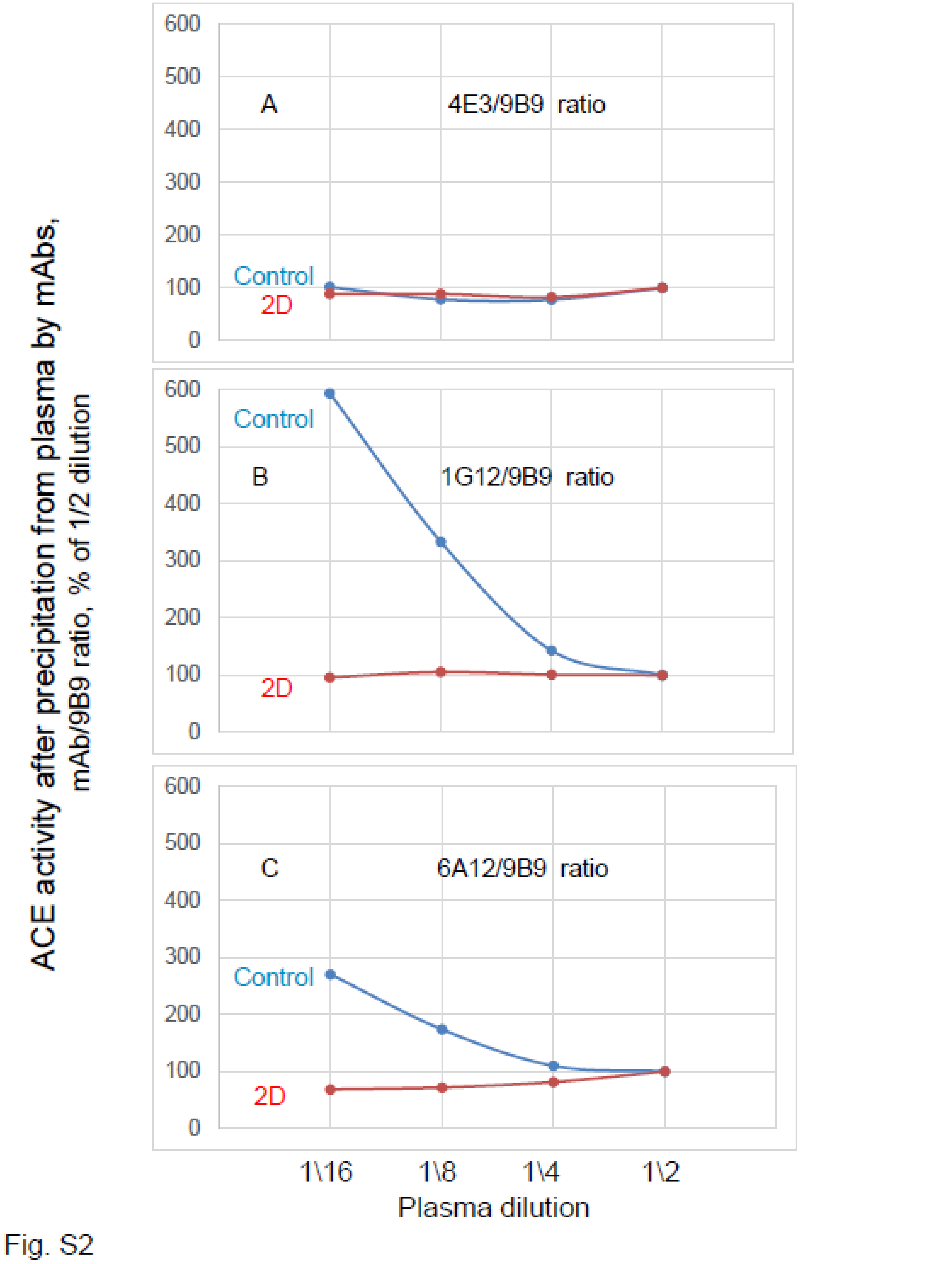

Figure S2. Effect of plasma dilution on mAbs binding to plasma ACE. Plasma from donor 2D (and control) were serially diluted and ACE activity precipitation by 4 mAbs were measured. ACE precipitation from both control and 2D plasmas by mAbs 9B9 (to the N domain of ACE) and by mAb 4E3 (to the C domain of ACE) was gradually (and similarly) decreased with dilution, which was reflected by identical values of 4E3/9B9 ratio at all dilutions (**A**). Binding of mAbs 1G12 (**B**) and 6A12 (**C**) to ACE from control plasma significantly increased with dilution (relative to the binding of mAb 9B9), indicating that blood component, bound to ACE, dissociated from the complex with ACE with dilutions which led to unmasking epitopes for these mAbs, whereas relative binding of mAbs 1G12 and 6A12 to ACE prom plasma 2D did not increase with dilution, but even decreased. Data, presented as mAb/9B9 ratios, are expressed as % (mean of triplicates) from dilution 1/2. (SD of triplicates were less than 10%).

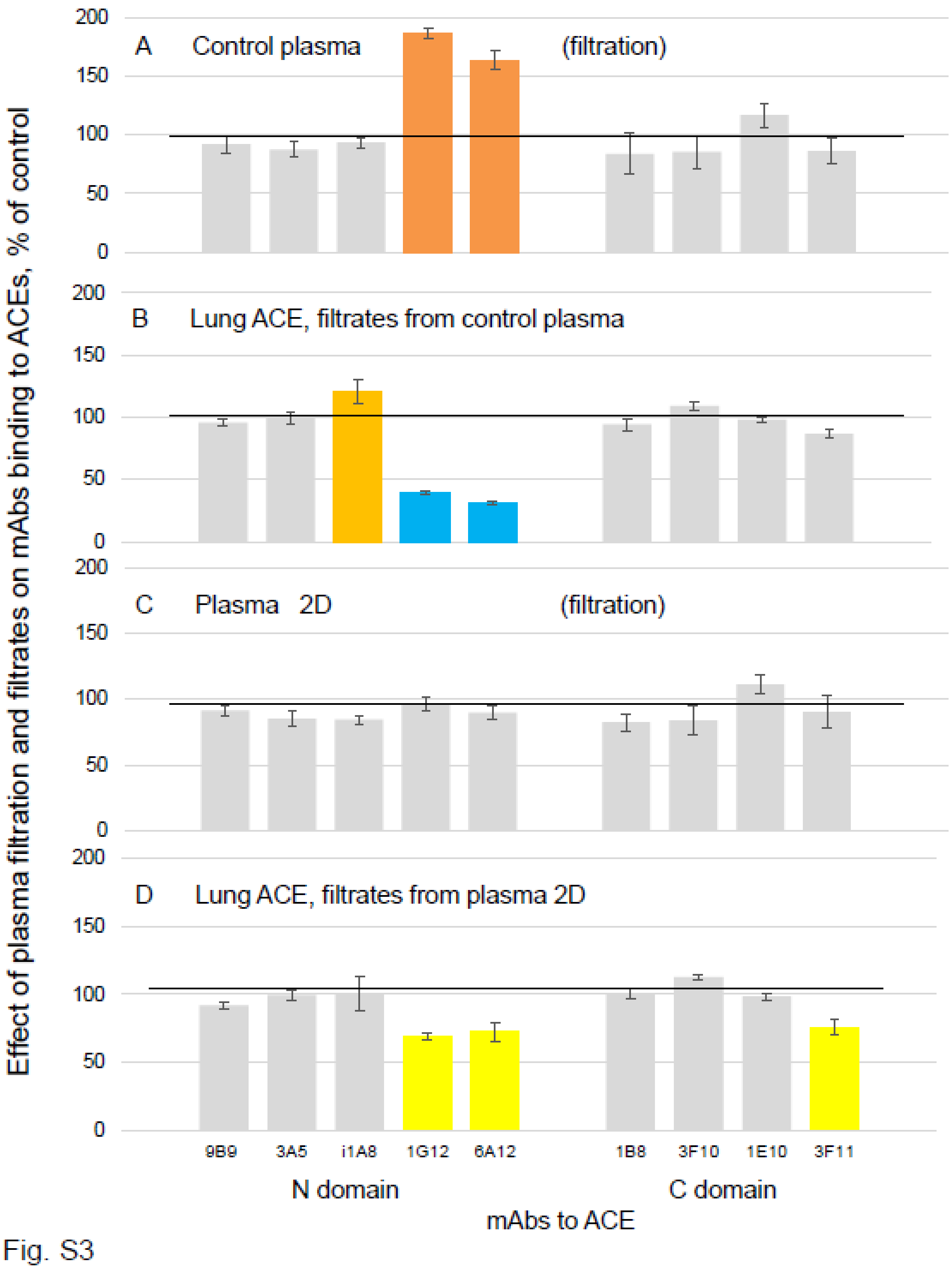

Figure S3. Effect of plasma filtration and plasma filtrates on mAbs binding to ACEs. Control and donor 2D plasmas (2 ml) were filtered (by centrifugation) through filters with 3, 10, 30, and 100 kD pores. Plasmas were concentrated 10-folds and then diluted 10-fold to initial volume. ACE activity precipitation from restored plasmas (at 1/5 dilution) by different mAbs was measured (**A** and **C**) as in Fig.1-2. Filtrates (at 90% concentration) were added to purified lung ACE (final ACE activity about 10 mU/ml), and ACE activity precipitation by different mAbs (**B** and **D**) was measured as in **A**,**C**. Data are expressed as mean value of precipitated ACE activity from 4 filtrates from 4 filters (mean ± SD) from control (PBS instead of filtrates) by each mAb.

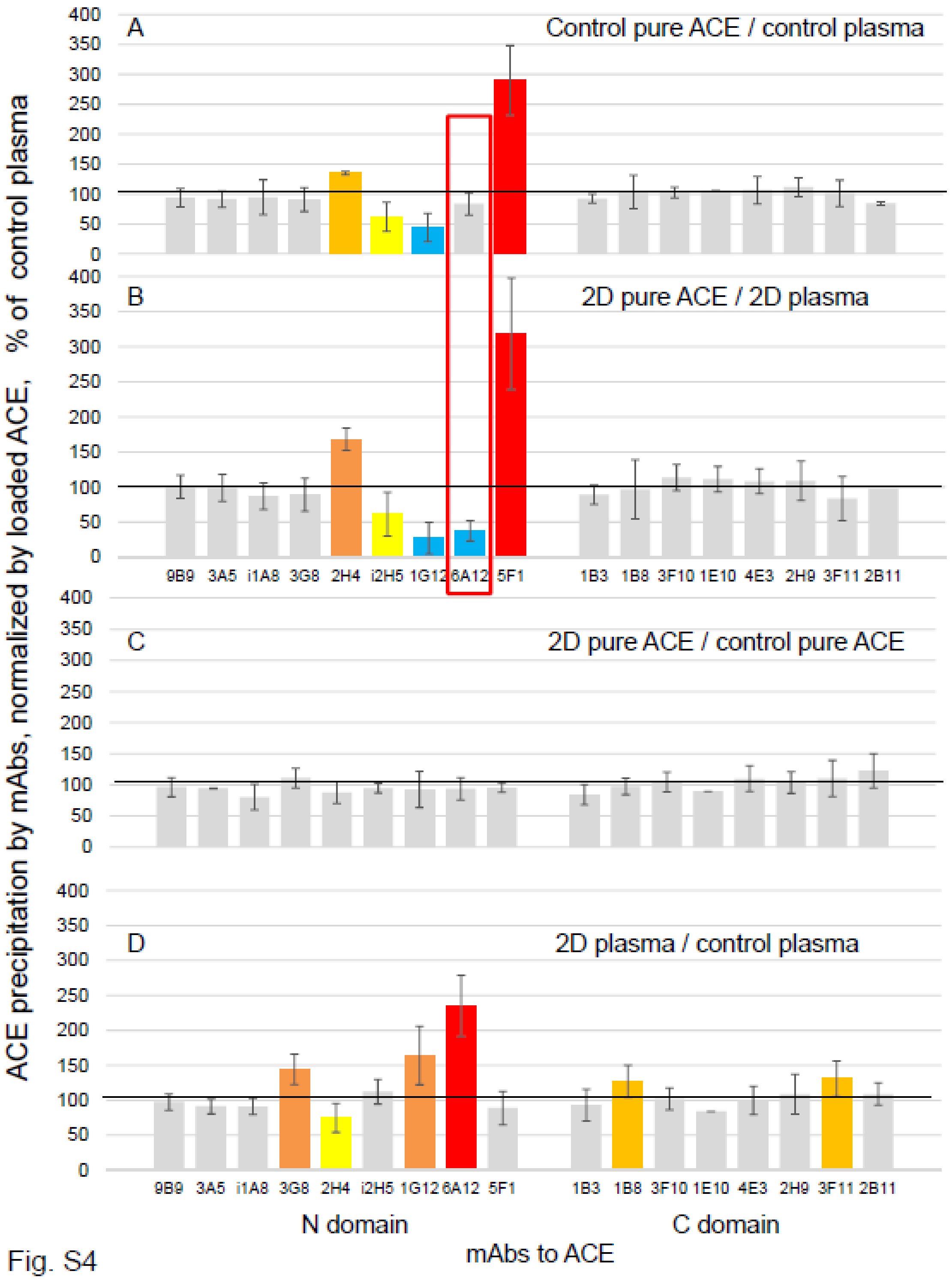

Figure S4. Effect of ACEs purification from control and 2D plasmas on mAbs binding. Filtered (0.45 µM) control and 2D plasmas (5 ml) were incubated overnight with 5 ml of lisinopril-Sepharose and then loaded to empty 10 ml column with filter. Flow-through fraction was collected and then column was washed 10 times by 5 ml portions of PBS (till optical density of washing solution became zeroed). ACE was eluted from column by six cycles of 2 ml portions of 50 mM borate buffer, pH 9.5 (with 150 mM NaCl). Pooled active fractions (usually 2nd-3rd fractions with ACE activity about 100 mU/ml) were used for conformational fingerprinting using a set of 16 mAbs to ACE.

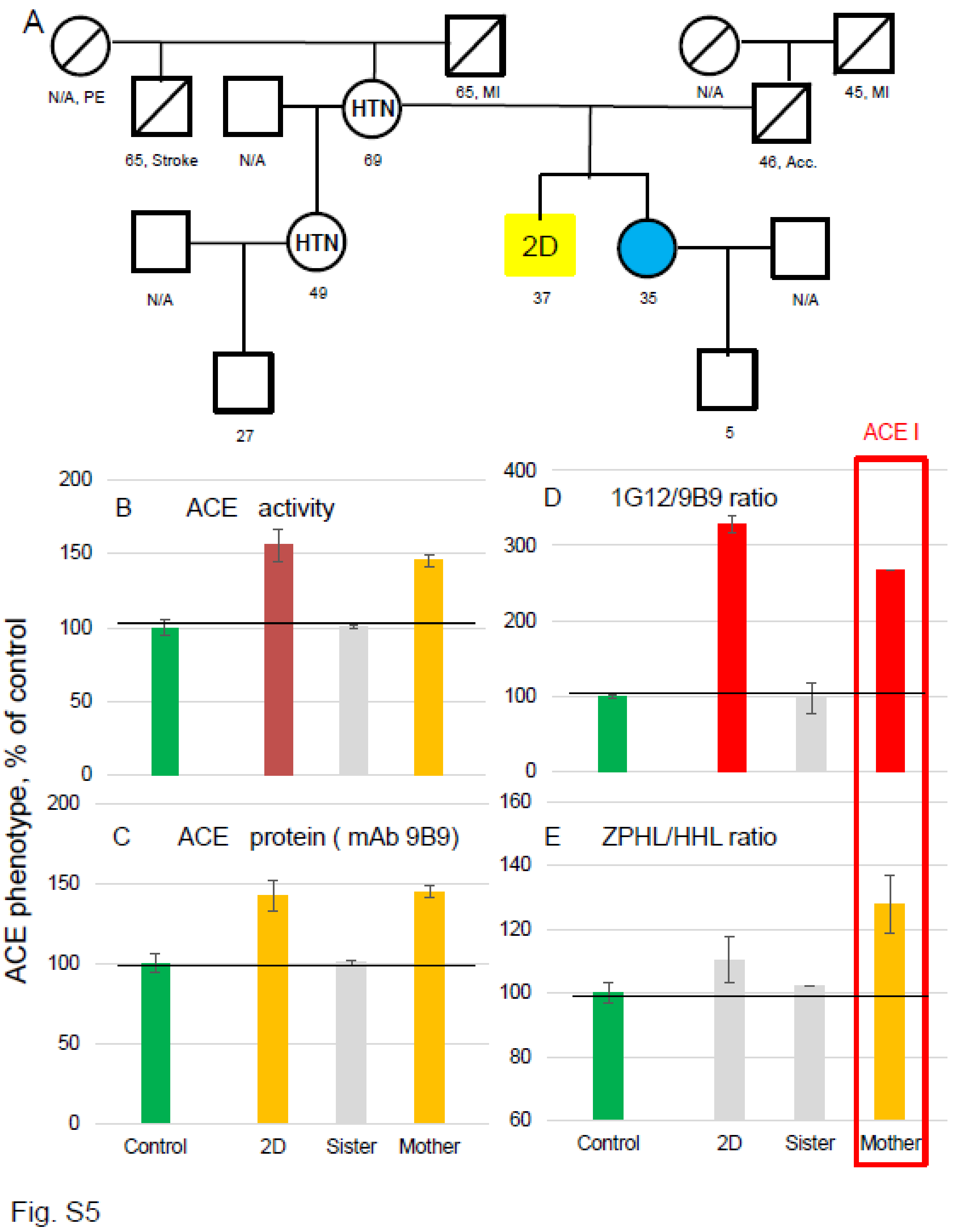

Figure S5. Family tree of donor 2D and plasma ACE phenotyping in his relatives.

A. Family tree with available data (age and diseases). **B-E**. Characteristics of ACE phenotype in donor 2D, his mother and sister are expressed as a % from that in control (pooled) plasma.

The evidence for the presence of exogenous ACE inhibitor in donor's mother plasma is red boxed. Based on this data sister's DNA was chosen as a negative control for DNA sequencing.

File S1.Sequencing.

1. Sanger sequencing Genomic DNA was obtained from whole blood of donor 2D (and his sister) by QIAamp DNA Mini Kit (Qiagen, Valencia, CA), and 6 exons of ACE gene (*ACE*, 7-11th and 13th), were amplified and sequenced, using primers listed in Table 1. Two exons of lysozyme gene (*LYZL1*) - 2th and 4th, coding the most of amyloidogenic mutations were amplified and sequenced using primers, kindly provided by Dr. T. Prokaeva (Boston University, Boston, MA].

Table 1. Primers used for amplification and sequencing of ACE gene.

Exon	Sequence	Amplicon	Source
7	Fw: TCATGCACAGGGAGTTGACC<br>Rv: GGAACCCCAGAACGTGCTC	305 bp	Dufour et al. 2000
8	Fw: ATCCGTCACTCTCACCCTCG<br>Rv:AGGCAGAAAGAAGGGCAGAGC	364 bp	In-house
9&10	9 Fw: TCTTTCCTCTCCTTTCATCTC<br>10Rv: TTGAGGGTGTGACAGTACTG	604 bp	Dufour et al. 2000
11	CP29 (Fw): TCACACCCTCAATCCACTTCTC<br>CP30 (Rv): ATTTGTGTCGCCCCATGCCAG	573 bp	SeqWright, Inc. (Houston, TX)]
13	CP33 (Fw): AAGGTGGGTCCTCAACTCTGG<br>CP34 (Rv): GGTCTCACCAGAAGCCATTATC	606 bp	SeqWright, Inc. (Houston, TX)

Heterozygous reading (C/T) was obtained from genomic DNA sequence of the donor 2D in the 13th exon - SNP rs4316.

2. Sequencing of 6000+ clinically relevant genes:

Sequencing of the exomes of 6000+ clinically relevant genes of 2D patient demonstrated 1143 substitutions in 908 genes in 2D (and 1256 substitutions in 1063 genes in his unaffected sister) in comparison to reference genome. We had a priority hypothesis that patient 2D may have a mutation (s) associated with bilirubin metabolism and therefore, at first, we paid attention to such suspicious mutations. We did not find rare mutations/variants in *BVLRA* (biliverdin reductase A) and *BVLRB* (biliverdin reductase B), therefore, appearance of optical isomers of bilirubin that may associate with mutations of these genes in patient 2D unlikely. We also did not find mutations in *UGTA1* (UPPglucoronosyltransferase family 1 member A1 (main enzyme responsible for glucuronidation of bilirubin.

Among other remaining suspicious mutations/polymorphic variants, two heterozygous variants that are present in donor 2D and absent in his unaffected sister should be mentioned; *UGT1A3*: pTrp11Arg (with high frequency in population, between 20-50%, and *UGT2A2*:p.Gly317Arg with frequency between 7 and 10% (Table S2)). Each variant alone is hardly be capable to affect bilirubin metabolism, but its combination (with frequency of less than 2%) may play some role.

We also mentioned two polymorphic variants of *ABCC2*, but the frequency of these variants in population is also high, ruling out these variants as causal. We also found mutation of *HBD* pAla28Ser (associated with deltathalassemia and with low frequency (0.21%) and *SLC4A1* pGlu40Lys, which is defined as definitively pathogenic for spherocytosis of the 4th type. Patients with *SLC4A1* mutations had the lowest levels of unconjugated bilirubin.

The first candidate for causal mutation in patient 2D is likely very rare mutation in *ABCG2* (p Met71Val) –gene coding for membrane transporter ABCG2, which participates in transport of more than 200 compounds, including bilirubin. The same mutation was described recently (Zambo et al. 2018) and was shown to lead to decreased surface expression of ABCG2, but influence of this mutation on bilirubin metabolism was not analyzed yet.

Table S2. The list of suspicious substitutions in genes of donor 2D

##	Gene	Substitution	Frequency, %
1	*ABCC2*	Val417Ile	19.0
2	*ABCC2*	Val1188Glu	4.5
3	*ABCC2*	Cys1515Tyr	5.5
4	*ABCG2*	Met71Val	**0.08**
5	*HBD*	Ala28Ser	**0.21**
6	*SLC41A1*	Glu401Lys	10.7
7	*SLCO1B1*	Asn130Asp	29.8
8	*UGT1A3*	Trp11Argr	20-50
9	*UGT2A2*	Gly317Argr	7-10

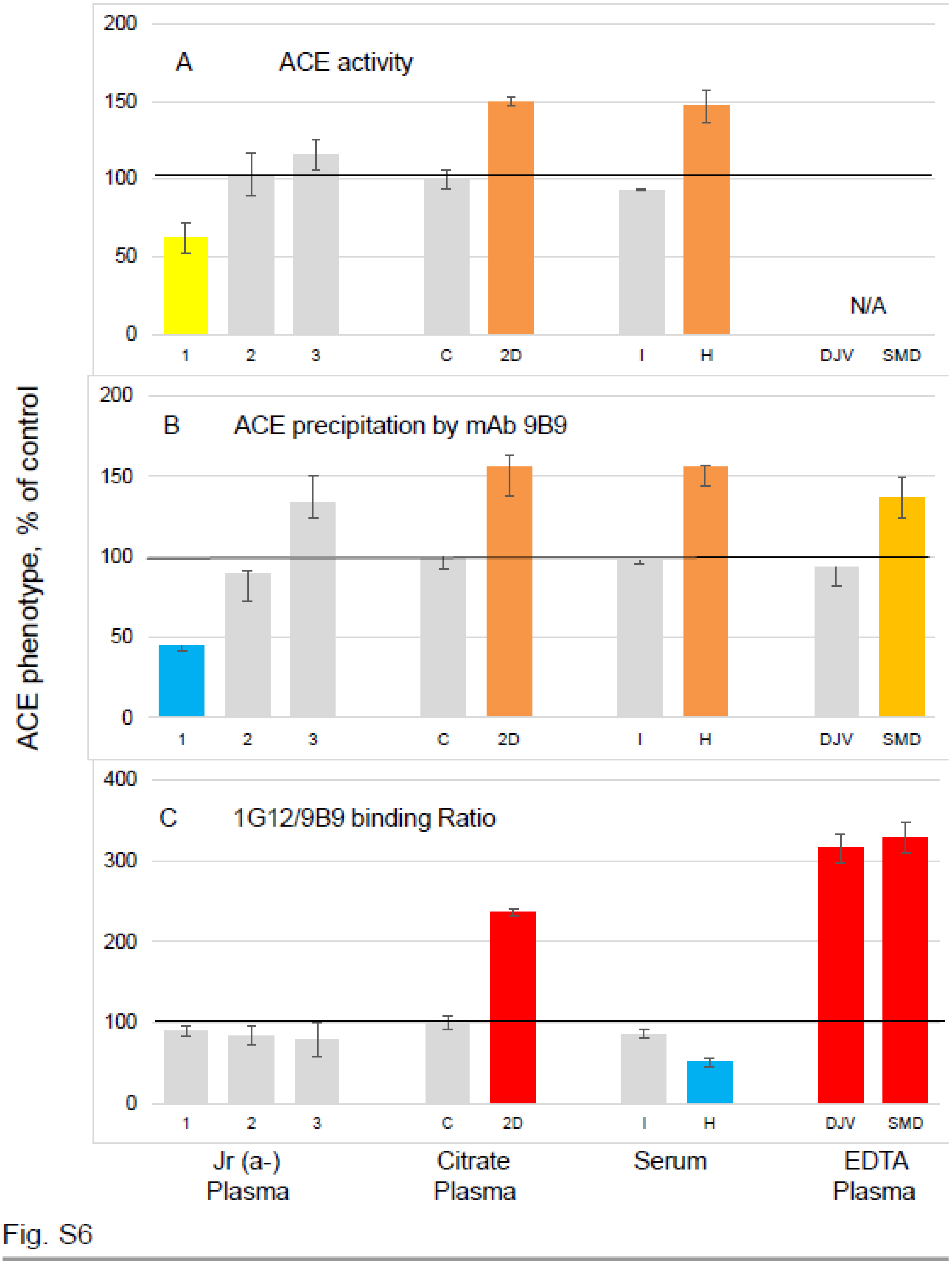

Figure S6. ACE phenotyping in ABCG2 null patients.

Plasma samples from three patients with *ABCG2* null mutants (Zelinski et al. 2012) were provided by Dr. C.M. Westhoff (Immunohematology and Genomics and National Center for Blood Group Genomics, New York Blood Center, NY). ACE activity **(A)**, amount of immunoreactive ACE protein determined with mAb 9B9 **(B)** and 1G12/9B9 binding ratio **(C)** were quantified in the plasma of tested patients and presented as in Fig.1. As a negative control, we used pooled plasma sample from healthy donors considered as 100%), and as a positive control (for conformationally changed ACE) we used plasma of donor 2D. As an additional control, we used serum and EDTA plasma from two donors (I and H) and volunteers (laboratory personnel-DJV and SMD). Data are expressed as mean value of ACE phenotype characteristics (mean ± SD) from control.

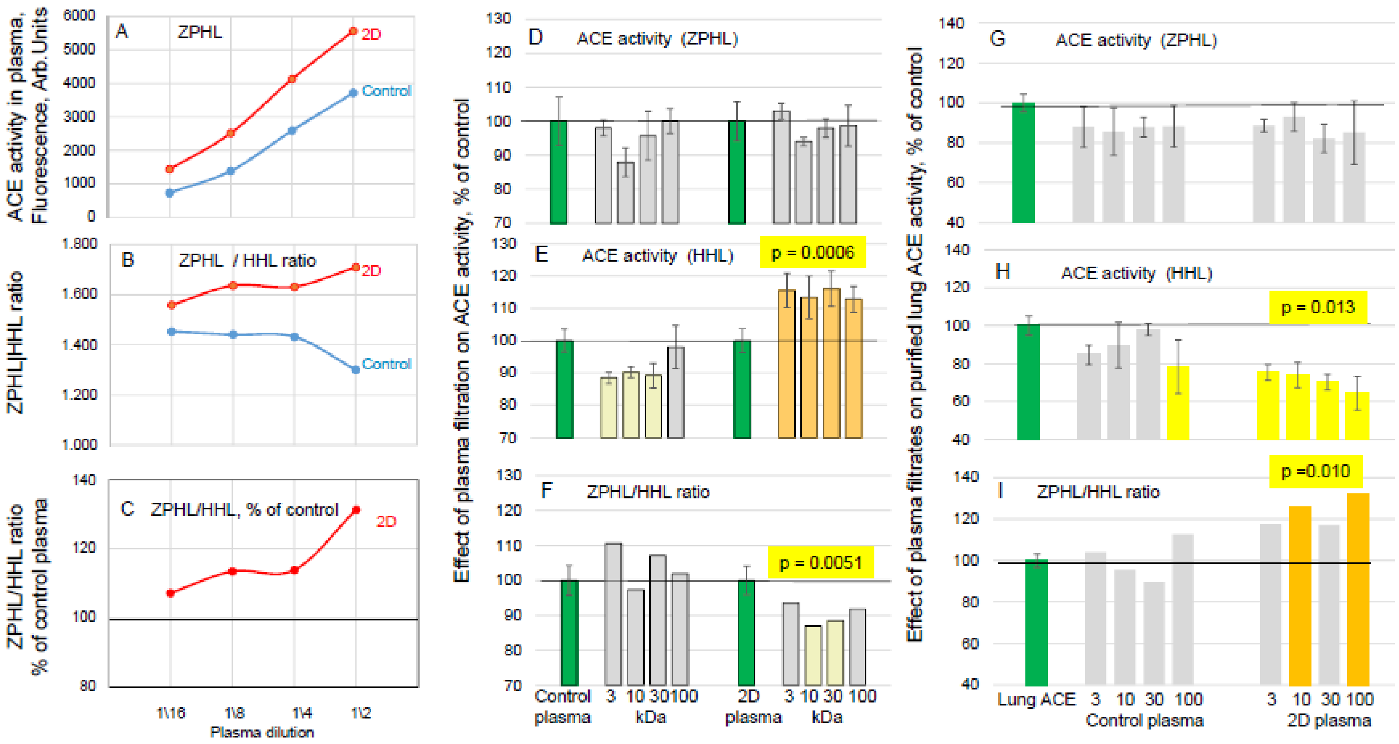

Fig. S7

Figure S7. Effect of plasma dilution, filtration and plasma filtrates on ACE activity.

Plasma from donor 2D and control plasma were serially diluted (as in Fig.S2) or filtered (by centrifugation) through filters with 3, 10, 30, and 100 kD pores. Undiluted plasmas which were concentrated 10-fold were then diluted 10-fold to initial volume. Filtrates (at 90% concentration) were added to purified lung ACE (final ACE activity about 10 mU/ml). ACE activity in diluted plasmas **(A-C)** and filtered plasmas **(D-F)** or in the solution of purified lung ACE in the presence of plasma filtrates **(G-I)** was measured as in Fig.1 Data are presented as mean value of ACE activity (in triplicates) with ZPHL **(A)**; mean value of ZPHL/HHL ratios **(B)**; % from dilution 1/2 **(C)**; % from unfiltered plasmas **(D-F)**; % of ACE activity in the presence of PBS instead of filtrates **(G-I)**. Coloring is as in Fig.1 and 2

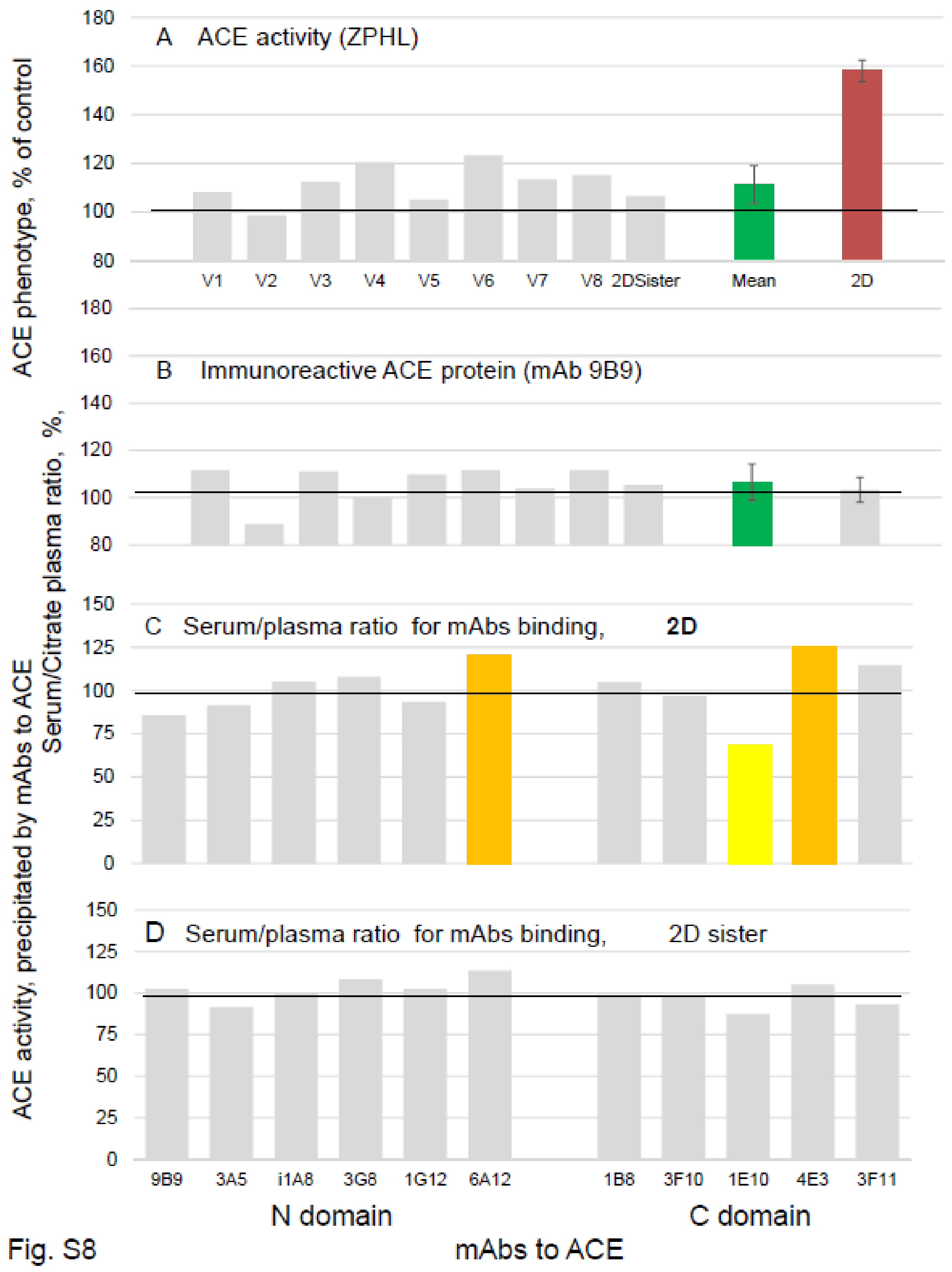

Figure S8. ACE phenotyping in plasma and serum.

A-B. Plasmas from 8 volunteers, donor 2D and his sister were phenotyped for ACE. ACE activity with ZPHL **(A)** and the amount of immunoreactive ACE protein determined with mAb 9B9 **(B)** were quantified as in Fig.1. Data are presented as mean of triplicates (with SD less than 10% for individual volunteers and (mean $\pm$ SD) for values from 9 volunteers (green bar) and (mean $\pm$ SD) for two experiments (each in triplicates) for donor 2D.

C-D. ACE activity from serum and citrated plasma of donor 2D **(C)** and his sister **(D)** was precipitated by 11 tested mAbs to ACE. Data were presented as serum/plasma binding ratio for each mAb (which were based on ACE activity precipitation in serum and in plasma in triplicates).

File S2. Clinical characteristics of patients with Gilbert and Crigler-Najjar syndromes.

Gilbert syndrome. 1. Patient 1 (2 y.o), total bilirubin 24.6 µM; 2. Patient 2 (8 y.o), total bilirubin 32 µM. Both patients had 7TA/7TA genotype of UGT1A1*28 polymorphism

Crigler-Najjar syndrome. Both patients are adults > 30 years old, but we could not give the year of birth due to privacy protection- other will know who they are because this disorder is very rare.

1. Patient 1- total bilirubin 240 µM; direct bilirubin could not be determined because of icteric sample; albumin was 53 mg/ml.
2. Patient 2 - total bilirubin 147 µM; direct bilirubin was 13 µM, i.e. 8.8%; albumin – 47 mg/ml

Both patients are CN type 2 and both have the same genotype: c.44T>G.

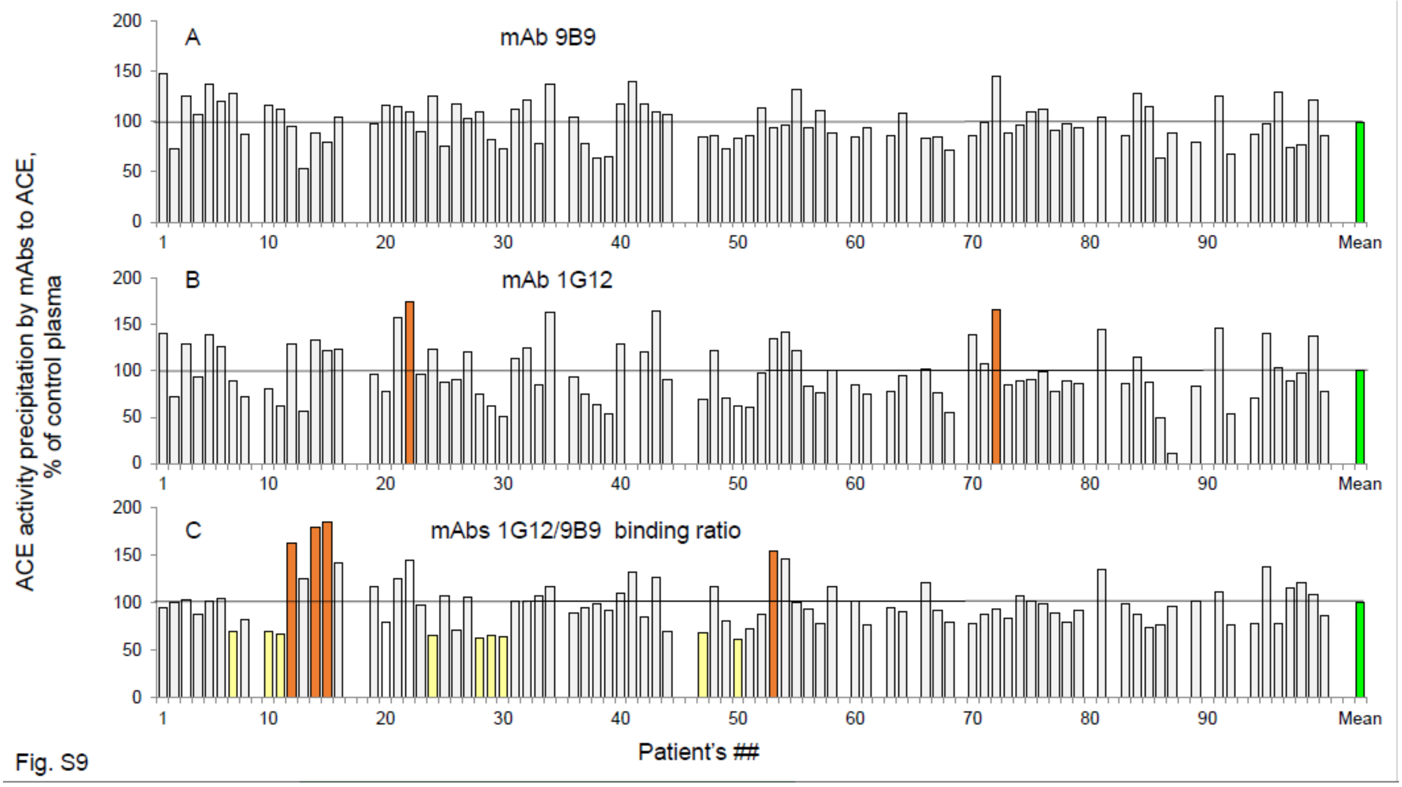

Figure S9. ACE local conformation (Gilbert syndrome carriers?).

ACE activity precipitated by mAbs 9B9 (**A)** and 1G12 (**B**), as well as 1G12/9B9 ratio (**C)** from 85 plasma samples of unrelated individuals, were quantified as in Fig. 1. Data are presented as % of ACE activity or 1G12/9B9 ratio from that for pooled plasma samples from healthy volunteers. Bars highlighted with yellow - samples with 1G12/9B9 ratio lower than 80%, bars highlighted with brown –-higher than 150% of control (mean value of 1G12/9B9 ratio in healthy population).
